# Development of an Engineered Bacterial Endophyte: Promoting Plant Growth Through Pyrroloquinoline Quinone (PQQ) Synthesis

**DOI:** 10.3390/microorganisms13020293

**Published:** 2025-01-28

**Authors:** Ti Fang, Shou-Chen Lo, Yu-Ning Yu, Nga-Lai Sou, Shih-Hsun Walter Hung, Jian-Hau Peng, En-Pei Isabel Chiang, Chieh-Chen Huang

**Affiliations:** 1 Department of Life Science, National Chung Hsing University, Taichung 402, Taiwan; quinnfang@hotmail.com (T.F.); scl@dragon.nchu.edu.tw (S.-C.L.); aoy92106@gmail.com (Y.-N.Y.); walter030170@gmail.com (S.-H.W.H.); 2 Biotechnology Program of Industry-Academia Collaboration, National Chung Hsing University, Taichung 402, Taiwan; 3 Department of Food Science and Biotechnology, National Chung Hsing University, Taichung 402, Taiwan; looksusan2013@gmail.com (N.-L.S.); jianhau.peng@gmail.com (J.-H.P.); chiangisabel@nchu.edu.tw (E.-P.I.C.); 4 Innovation and Development Center of Sustainable Agriculture (IDCSA), National Chung Hsing University, Taichung 402, Taiwan; 5 Advanced Plant and Food Crop Biotechnology Center (APFCBC), National Chung Hsing University, Taichung 402, Taiwan; 6 Institute of Plant and Microbial Biology, Academia Sinica, Taipei 115, Taiwan; 7 Doctoral Program in Microbial Genomics, National Chung Hsing University, Taichung 402, Taiwan

**Keywords:** PQQ, pyrroloquinoline quinone, engineered endophyte, *B. subtilis*, biostimulants, growth promotion

## Abstract

Endophytic bacteria are a group of microorganisms that can intercellularly colonize plant hosts without causing apparent damage or disease. Our previous works found that a pyrroloquinoline quinone (PQQ)-producing endophyte could promote plant growth and systemic tolerance. To demonstrate this PQQ-producing endophyte’s beneficial role in plants, a set of five PQQ synthesis genes from *Gluconobacter oxydans* was introduced into both *Escherichia coli* JM109 and *Bacillus subtilis* RM125, a BsuM-deficient mutant of laboratory strain *B. subtilis* 168. Interestingly, both strains harboring the PQQ synthesis genes exhibited significantly higher optimal optical density than control strains. In a carbon flux analysis, both strains showed a noticeable increase in their citric acid, alpha-ketoglutaric acid, and succinic acid levels. Conversely, in *E. coli*, pyruvic acid, malic acid, and fumaric acid levels decreased. These results suggest that PQQ impacts various host species differently. Furthermore, the presence of PQQ in fermentation broth was also confirmed in the RM125 PQQ synthesis recombinant strain. Subsequent experiments by inoculating those *Bacillus* strains revealed that the laboratory host strain could function as an endophyte, and the PQQ transgenic strain could further promote the growth of *Arabidopsis thaliana* and increase the number of siliques. These findings confirm PQQ’s vital role in endophyte-mediated plant growth promotion and also suggest the potential of *B. subtilis* transformed with PQQ genes as an engineered endophyte for studying PQQ’s biological functions in plants. This research is a step forward in understanding how specific substances can beneficially influence plant growth and systemic tolerance through endophytic mechanisms.

## 1. Introduction

Plant endophytes are microorganisms that reside within plant tissues and do not usually cause disease. This association confers numerous benefits to plants, including disease resistance, stress tolerance, and enhanced nutrient absorption efficiency [1].

Consequently, plant endophytes are regarded as potential “biostimulants” capable of improving plant performance and yield. Plant endophytes exhibit broad prospects for applications in crop production, contributing to increased growth efficiency and reduced reliance on chemical fertilizers and pesticides, aligning with the expectations of sustainable agriculture [2].

Applying plant endophytes as microbial biofertilizers offers multiple advantages and holds exciting prospects [3]. Plant endophytes facilitate nutrient absorption in plants, in contrast to traditional chemical fertilizers that directly provide supplementary nutrients. This enables the use of endophytic biofertilizers to enhance plants’ utilization efficiency for both organic and inorganic nutrients in the soil, thereby reducing dependence on chemical fertilizers [4]. This can not only mitigate soil and water pollution but also contribute to maintaining soil ecological balance and minimizing the spread of pathogens in the soil [4]. These positive impacts on soil health and ecosystem stability align with the principles of sustainable agriculture, promising long-term agricultural sustainability and improved agricultural production efficiency.

On the other hand, plant endophytes may also play a crucial role in assisting plants in resisting diseases. Endophytes typically form symbiotic relationships with plant roots, occupying space in those roots and the rhizosphere [5]. This occupation can impede pathogen invasions, reducing the opportunities for pathogens to come into contact with the plant [6]. The competition between endophytes and pathogens is referred to as competitive exclusion, contributing to maintaining plant health [7]. Some endophytic microorganisms can produce antibiotics that exhibit inhibitory or lethal effects against plant pathogens [8]. Furthermore, endophytes can systematically activate a plant’s immune system [6].

In previous studies, a plant endophyte, *Burkholderia seminalis* 869T2 whose previous name was *Burkholderia cenocepacia* 869T2, was identified that assists bananas in resisting yellow leaf disease [9] and enhances the growth efficiency of various crops [2]. Through further research, it was discovered that *B. seminalis* 869T2 can secrete a small molecule composed of two amino acids with a molecular weight of 330 Da, which is believed to be PQQ (pyrroloquinoline quinone). Plant-beneficial microbes can produce metabolites that promote plant growth, such as growth hormones (auxins), steroid hormones (gibberellins, GAs), and others [2]. With genome sequencing, 869T2 was found to possess gene sequences involved in biosynthesizing PQQ [2,9,10]. PQQ is an enzyme cofactor associated with lactate, glucose, alcohol, and methanol dehydrogenases [11,12,13,14]. Recent studies have indicated that PQQ indirectly influences several energy metabolism pathways [15,16]. The rhizosphere bacterium *Pseudomonas fluorescens* B16 plays a vital role in stimulating plant growth through PQQ [15]. Besides applications in agriculture, research on PQQ is thriving, with numerous studies indicating its effectiveness in various fields. These include Parkinson’s disease [17], the fracture-healing process [18], athletic performance [19], and piglet survival rates [20], among others. This extensive range of functional effects highlights the broad utility and significant research value of PQQ.

On the other hand, *Gluconobacter oxydans* 621H, a Gram-negative soil bacterium, is recognized for its PQQ synthesis genes (Supplemental Figure S1). *Gluconobacter oxydans* has a relatively short PQQ gene cluster, including *pqqA*, *pqqB*, *pqqC*, *pqqD*, and *pqqE*, among the sequenced species [10]. Previous studies have demonstrated that exogenous PQQ synthesis genes from *G. oxydans* 621H remained functional when expressed in *Escherichia coli* JM109 [10]. Therefore, it is feasible to directly synthesize the PQQ gene cluster of *G. oxydans* for heterologous gene expression. Building upon these findings, the T7 promoter was replaced with the pR promoter, as a shuttle vector design to express genes both in *E. coli* and *B. subtilis* [21]. In this study, *E. coli* JM109 (DE3) and a well-known laboratory strain *B. subtilis* RM125 were used as hosts; thus, the T7 and pR promoters were chosen, respectively. Along with the growth of the transformed bacteria, metabolites related to amino acid synthesis and the tricarboxylic acid (TCA) cycle were analyzed. PQQ production was confirmed using LC-QTOF. For further application as plant growth-promoting microorganisms, the bacteria and culture medium were inoculated into *Arabidopsis thaliana* plants.

## 2. Materials and Methods

### 2.1. Bacterial Strains and Plasmids

*E. coli* JM109 (DE3) and *B. subtilis* RM125 strains were obtained from BCRC (Bioresource Collection and Research Center, Hsinchu, Taiwan). Previous studies have indicated that the hosts of phage F1 include *E. coli* and *B. subtilis*. Therefore, the plasmids containing the origin of replication (ori) of phage F1 were used for subsequent experiments, providing more options for future research [22]. The PQQ synthesis genes were optimized and then transferred into the *E. coli* JM109 (DE3) and *B. subtilis* RM125 host strains. The optimization was performed by AllBioLife Corp. (Taichung, Taiwan). AllBioLife Corp. (Taiwan) was commissioned to synthesize the PQQ synthesis gene cluster from Gluconobacter oxydans into the pET-28a(+) vector, with the T7 promoter replaced by the pR promoter. Consequently, two plasmids were utilized in the study: pET-28a-T7-PQQ for *E. coli* JM109 (DE3) and pET-28a-pR-PQQ for *B. subtilis* (Supplemental Figures S8 and S9; the DNA sequences of the gene cluster were included in Supplementary Materials). The transgenic strains were cultured in lysogeny broth (LB), and antibiotics were added to the transplanted strains; kanamycin (30 µg/mL) was used to cultivate transformants of *E. coli* and *B. subtilis.* Bacterial growth was measured by monitoring the optical density at 600 nm (GeneQuant 1300, GE Healthcare, Little Chalfont, Buckinghamshire, UK).

### 2.2. Escherichia coli Transformation

The competent cells of *E. coli* JM109 were prepared according to a previous study [23] with modifications. *E. coli* incubated in 5 mL of LB at 37 °C overnight. After the overnight incubation, it was sub-cultured in fresh LB at 37 °C for 3–6 h. *E. coli* cells were then placed on ice for 10 min, followed by centrifugation at 2500× *g* for 10 min at 4 °C. The supernatant was removed, and the pellet was resuspended on ice for 10 min in 1 mL of TB buffer (10 mM HEPES, 55 mM MnCl_2_, 15 mM CaCl_2_, 250 mM KCl, pH 6.7). The *E. coli* cells were then pelleted again via centrifugation at 2500× *g* for 10 min at 4 °C. The supernatant was removed, and the pellets were resuspended in 100 μL of TB buffer. The transformation procedure was performed as previously described [23]. Plasmids were mixed with competent cells and incubated on ice for 30 min. The cell-plasmid mixture was then heat-shocked at 42 °C for 30 s, followed by a 2 min incubation on ice. Afterward, 0.8 mL of SOC medium was added, and the transformed cells were incubated at 37 °C with shaking at 120 rpm for 1 h. Transformed cells were selected on LB agar plates containing kanamycin (30 µg/mL) and incubated at 37 °C. Plasmid-containing colonies were confirmed by randomly selecting colonies and extracting the plasmids.

### 2.3. Bacillus subtilis Transformation

The two-step culture method developed by Anagnostopoulos and Spizizen was used to prepare competent *B. subtilis* cells and transformation based on a nutrient-deficient method [24]. First, 1 L of 10x Spizizen salts was prepared (K_2_HPO_4_ (anhydrous), 140 g; KH_2_PO_4_ (anhydrous), 60 g; (NH_4_)_2_SO_4_, 20 g; trisodium citrate-2H_2_O, 10 g), 50% (*w*/*v*) glucose, 2% (*w*/*v*) MgSO_4_-7H_2_O, 2% (*w*/*v*) casamino acid, 5 mg/mL tryptophan, 5 mg/mL arginine, 5 mg/mL leucine, and 5 mg/mL threonine. Second, the amino acids were sterilized by passing through a 0.22 µm disposable filter, while the others were sterilized by autoclave. Then, 100 mL of TF I (10x Spizizen, 10 mL; 50% glucose, 1 mL; 2% MgSO_4_-7H_2_O, 1 mL; 2% casamino acid, 1 mL; 5 mg/mL tryptophan, 1 mL; 5 mg/mL, arginine, 1 mL; 5 mg/mL leucine, 1 mL; 5 mg/mL threonine, 1 mL) and TF II (10x Spizizen, 10 mL 50% glucose, 1 mL; 2% MgSO_4_-7H_2_O, 1 mL; 2% casamino acid, 0.5 mL; 5 mg/mL tryptophan, 0.1 mL; 5 mg/mL, arginine 0.1 mL; 5 mg/mL leucine, 0.1 mL; 5 mg/mL threonine, 0.1 mL) were prepared. The difference between TF I and TF II was the reduced amino acid content, which causes *B. subtilis* to actively uptake the exogenous plasmid. The transformation procedure was as follows: the *B. subtilis* was pre-cultured in LB for 15 h and was transferred 900 μL of TF I, 25 μL of 2% casamino acid, and 50 μL of the pre-cultured bacterial solution, and incubated at 37 °C for 3 h and 45 min. In total, 100 μL of TF I bacterial solution was transferred to 900 μL of TF II, and incubated at 37 °C for 1 h and 15 min. Upon completion, 100 μL of the TF II bacterial solution was transferred to a new tube, we added 5~6 μL of the plasmid to be transformed, and incubated it at 37 °C for 1 h. Finally, 300 μL of LB was added to the transformed bacterial solution and incubated it at 37 °C for 1 h. The culture was evenly spread onto LB plates containing 30 µg/mL kanamycin for selection. The sequencing and plasmid verification were performed by AllBioLife Corp. (Taichung, Taiwan).

### 2.4. Determination of Cellular Protein and Free Amino Acids

The *E. coli* and *B. subtilis* were cultured in 5 mL of LB medium with 0.5% glucose (1-13C glucose, 50% replace) in 20 mL tubes. For the *E. coli* experiments, samples were collected at 6, 18, 30, and 44 h, while for the *B. subtilis* experiments, samples were collected at 3, 6, 9, and 12 h. The amino acid standards, including homocysteine, were obtained from Sigma–Aldrich (St. Louis, MO, USA). Perchloric acid was acquired from J.T. Backer (Center Valley, PA, USA). An alga-derived amino acid mixture (U-13C, 97–99%; U-15N, 97–99%), DL-homocysteine (3,3,4,4-D4, 98%), and DL-cystathione (3,3,4,4-D4, 98%) were purchased from Cambridge Isotopic Laboratories (Tewksbury, MA, USA) and used as internal standards for free amino acid quantitation analysis. The collected *E. coli* and *B. subtilis* samples were sonicated, followed by the addition of 100 μL of ice-cold 0.4 M perchloric acid (PCA) for thorough mixing. The mixture was then centrifuged at 4 °C, 12,000× *g* for 30 min to precipitate the protein. The resulting solution was transferred to a microcentrifuge tube and subjected to vacuum freeze-drying. Subsequently, 300 μL of 2 M acetic acid and 600 μL of ddH_2_O were added to each sample and vortex-mixed. The sample was applied to a column containing 300–500 μL of cation exchange resin. After the column was washed with 1–3 mL of NH_4_OH, the eluate was collected in a glass tube and vacuum freeze-dried. Next, heptafluorobutyric propyl ester derivatives were prepared according to the following steps: each tube was supplemented with 500 μL of 1-propanol and 100 μL of CH_3_COCl, filled with nitrogen gas, and sealed. After thorough vortex-mixing, the tube was placed in a dry bath at 110 °C for 20 min. Subsequently, the tube was cooled to room temperature for a few minutes and dried with nitrogen gas. Then, 100 μL of heptafluorobutyric anhydride was added for the derivatization of the dried sample. The sealed tube filled with nitrogen gas was incubated at 60 °C for 20 min. After derivatization, the tube was cooled to room temperature and dried with nitrogen gas, and the heptafluorobutyric propyl ester derivatives were dissolved in 100 μL of ethyl acetate. The resulting solution was transferred to glass inserts for analysis using gas chromatography–mass spectrometry (GC-MS). To hydrolyze the protein pellet, 6 N HCl was added, followed by vacuum hydrolysis. Amino acids were then purified using cation exchange resin. The amino acids were converted into heptafluorobutyric propyl ester derivatives—as described in the method for free amino acid quantification—and separated on an HP-5MS column (inner diameter, 0.25 mm; length, 30 m; coating thickness, 0.25 μm). Isotopic enrichment was measured in electron capture negative ionization mode with a GC-MS instrument consisting of a 6890 GC and a 5975 MS (Agilent, Palo Alto, CA, USA), as described previously [25,26].

### 2.5. Determination of Tricarboxylic Acid Cycle (TCA Cycle) Metabolite Enrichment

*E. coli* and *B. subtilis* bacterial samples were thoroughly mixed with methanol and ice-distilled water at −20 °C. Chloroform was added to the mixture and vortexed for 10 min at 4 °C. Subsequently, the mixture was centrifuged for 10 min at 14,000× *g* and 4 °C, resulting in the formation of two distinct layers. The methanol and water layer contained the desired polar metabolites, including TCA-related metabolites. The desired metabolites included pyruvate, lactate, α-ketoglutarate, succinate, fumarate, malate, and citrate and were carefully transferred to a microcentrifuge tube, taking care not to disturb the middle layer. They were then dried using a freeze-dryer or vacuum centrifuge [27]. Each sample then underwent two steps of derivatization. Firstly, the dried precipitate was dissolved in 30 μL of pyridine containing 10 mg/mL methoxymation hydrochloride. The mixture was vigorously mixed for 30 s and heated at 70 °C for 30 min to methoxymate ketones (pyruvate and α-ketoglutarate). In the second step, 20 μL of N-tert-Butyldimethylsily-N-methyltrifluoroacetamide was added for the derivatization reaction, which proceeded for 1 h at 70 °C [28]. An Agilent 5975C GC-MS equipped with a DB-225MS (inner diameter: 0.25 mm; length: 30 m; coating thickness: 0.25 μm, Agilent, Palo Alto, CA, USA) column was used and maintained under the following GC conditions: carrier gas: helium; gas flow: 1.5 mL/min; inlet temperature: 240 °C; transfer line temperature: 240 °C; ion source temperature: 230 °C; the quadrupole was set at 150 °C. Data were collected using electron impact (70 eV). The oven program was started at 60 °C for 1 min, then the temperature was increased at a rate of 10 °C/min until 240 °C, and baked-out at 240 °C for 5 min [29].

### 2.6. Arabidopsis Cultivation

The cultivation method for *Arabidopsis thaliana* ecotype Columbia (Col-0) was provided by Chieh-Chen Huang and followed the protocols as previously described ([2], i.e., grown in soil at 24 to 26 °C with a 16 h light/8 h dark cycle). Briefly, the surface-sterilizing seeds were kept under 4 °C for at least 4 days for seed vernalization and then germinated in plug cells. After the seedlings reached the growth stage of 4 to 6 rosette leaves emergence, seedlings were transplanted into pots (diameter: 6.2 cm; depth: 8 cm) and grown in a plant growth room with controlled conditions: 21 ± 2 °C, RH 40 ± 5%, 14/10 day–night light period and light density 90–120 μmol/m^2^/s. All the seedlings were taken care of by the same watering frequency, and no fertilizer was used in this experiment. The plants were divided into four groups, each inoculated with either *B. subtilis* strain RM125 WT, *B. subtilis* strain RM125 PQQ, *B. seminalis* strain 869T2, or water (control) during the growth period between the development of the second and third leaf pairs. *B. subtilis* containing the PQQ gene and *B. subtilis* without the PQQ gene were cultured under the same conditions for 8 h and centrifuged to remove the supernatant to avoid excessive salinity affecting plant growth. The bacterial cells were resuspended with sterilized deionized water and the OD value was adjusted to 1. The bacterial solution was then appropriately irrigated in pots containing *A. thaliana*, at which point, the *A. thaliana* should have only produced its second pair of true leaves. Each experimental group has 10 plants (*n* = 10). The evaluation of the plant’s vegetative or reproductive growth at 21 days after inoculation (DAI 21) was performed by measuring a total of nine phenotypic parameters, including leaf length, leaf width, leaf rosettes, number of leaves, plant height, siliques, number of first inflorescences, number of second inflorescences, and number of third inflorescences.

### 2.7. Microbial Strain Validation

Two days after inoculation, the plants were washed with water and disinfected with an appropriate ratio of disinfectant solution. A mixture of 0.1% Tween 20^TM^ and a 30-fold diluted hypochlorous acid solution was prepared as a disinfectant solution. First, the plant tissue was surface-disinfected with 70% ethanol for 1 min and then rinsed with sterile water for 30 s. After that, the plant tissue was disinfected with the prepared disinfectant solution for 10 min. After disinfection, the disinfectant solution was discarded under sterile conditions, and the plants were washed four to five times with sterile ddH_2_O by shaking for 30 s each time. Then, the plants were crushed with forceps in microcentrifuge tubes, and 15 µL was taken and streaked onto LB agar plates after several 10-fold dilutions for strain identification. Genomic DNA from colonies that were able to grow in LB was extracted using the Tissue and Cell Genomic DNA Purification Kit (GMbiolab Co., Ltd., Taichung, Taiwan). The concentration of genomic DNA used in the 50 μL PCR reactions was prepared according to the manufacturer’s instructions (KOD-Plus-, TOYOBO, Tokyo, Japan). The species of the colonies were identified using 16S rRNA gene sequencing with the primer pair U1510R (5′-GGTTACCTTGTTACGACTT-3′) and E8F (5′-AGAGTTTGATCCTGGCTCAG-3′) [30]. Following PCR amplification, the samples were sent to AllBioLife Corp. (Taiwan) for sequencing. The resulting sequences were analyzed using the NCBI BLAST database to identify the bacterial species.

### 2.8. Extraction and Detection of PQQ

Preparing PQQ involves adding NaOH to facilitate its complete dissolution in deionized water, as pure PQQ has very low solubility in this substance. In this study, 2 µL of 10 N NaOH was added to a 2500 ppm PQQ solution to fully dissolve the PQQ. Subsequently, the solution was passed through a 0.22 µm filter and stored in a refrigerator.

PQQ extraction was performed using a modified version of a protocol described in a previous study [31]. The *B. subtilis* RM125 PQQ bacterial sample was mixed with 3 mL of ethyl acetate and vortexed for 5 min. The resulting mixture was subjected to centrifugation to separate the two phases. The lower phase was combined with 250 μL of 6 M HCl and 5 mL of ethyl acetate, followed by vortex-mixing. The mixture was then centrifuged to separate the phases, and the upper phase containing PQQ was collected. The collected upper phase was mixed with 500 μL of water, and the PQQ extract was dried using a vacuum centrifuge. The dried pellets were mixed with 5 mL of 0.1 M HCl and applied to a Sep-Pak C cartridge (Waters Corporation, Drinagh, Ireland) pre-equilibrated with 10 mL of 1 mM HCl. PQQ was eluted from the cartridge using 2 mL of 5% (*v*/*v*) pyridine–water, and the eluate was dried using a vacuum centrifuge. The dried pellets were dissolved in 500 μL of water.

PQQ analysis was conducted in negative ion electrospray ionization (ESI) mode using the Thermos Ultimate 3000 ultra-performance liquid chromatography (UPLC) system (Dionex/Thermo Fisher Scientific, Idstein, Germany) coupled with the amaZon speed mass spectrometer equipped with Compass Data Analysis software, Version 4.0 (Bruker, Billerica, MA, USA). The samples were separated on a C18 column (150 × 2.1 mm, 3 μm, GL Sciences, Inc., Torrance, CA, USA). The liquid chromatography mobile phase consisted of 10 mM dibutylammonium acetate as mobile phase A and acetonitrile as mobile phase B. The following LC gradient was employed: 0 to 6 min, 70% A; 6 to 12 min, decrease solvent A to 10%, followed by 10 min of equilibration before the next injection. The flow rate was set at 200 μL/min. Absorbance was monitored at 249 nm, 280 nm, and 422 nm using a diode array detector (DAD) chromatogram. The mass spectrometer was operated in multiple reaction monitoring (MRM) mode. The precursor ion of PQQ (*m*/*z* 329) was selected, and the product ions of PQQ (*m*/*z* 241 and *m*/*z* 285) were monitored. Electrospray ionization parameters were set as follows: dry gas flow rate of 9.0 L/min, nebulizer gas flow rate of 40 psi, dry temperature of 250 °C, and ionization voltage of −4500 V. For quadrupole time-of-flight (QTOF) analysis, a different column (ACQUITY T3 1.8 µm 100 × 2.1 mm) was used, and the flow rate was adjusted to 0.2 mL/min. The mobile phase consisted of A (ammonium acetate) and B (ACN). The gradient conditions were as follows: 98% A and 2% B from 0 to 1 min; 80% A and 20% B from 2 to 10 min; 50% A and 50% B from 11 to 15 min; 100% B from 16 to 18 min; and finally, from 18 min onward, 98% A and 2% B for 2 min.

### 2.9. Plasmid Retention in B. subtilis

The *B. subtilis* RM125 PQQ strain was routinely cultured in LB medium with kanamycin (30 µg/mL). To investigate the retention of plasmid in *B. subtilis* under non-antibiotic culture conditions, the bacteria strain was cultured in LB medium without kanamycin for 58 h. During this period, a portion of the bacterial culture was sampled for an additional growth test in LB medium without antibiotics, and CFU (colony forming unit) was counted on LB agar containing kanamycin. The OD values after 12 h of culture in the growth test were recorded and compared with those of the *B. subtilis* RM125 wild-type strain. CFU was determined by plate counting after serial dilution of bacterial samples with sterile ddH_2_ O and incubation at 37 °C overnight.

## 3. Results

### 3.1. Characterization of Transformed E. coli JM109/pET-28a-PQQ

**Figure 1:**
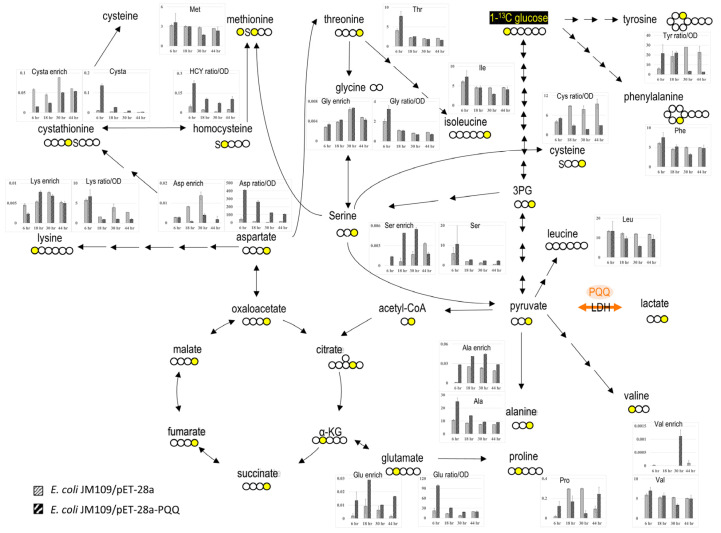
Expression of the PQQ pathway enhanced amino acid fluxes and production in *E. coli* JM109. After metabolism, radioactive glucose increased in tyrosine, phenylalanine, serine, alanine, proline, glutamate, aspartate, methionine, threonine, and some derivatives, including homocysteine and cystathionine. The labeled carbon is marked in yellow. The lactate dehydrogenase (LDH) that PQQ may act as cofactor is highlighted. Details will be provided in Supplementary Materials. The data are presented as the mean and standard deviation of two replicates.

After the pET-28a-T7-PQQ was transformed into *E. coli* JM109, the growth curve was examined and metabolic flux tracing techniques were used to understand the changes in *E. coli*. Figure S3 shows that the final optical density (OD) value of the JM109 strain containing the PQQ genes was twice that of the control group, indicating that PQQ genes effectively improve *E. coli*’s growth efficiency. To understand the reasons behind the growth rate changes, the amino acid profile and metabolic flux (Figure 1, for high-resolution images, see Figure S2a–c) inside the cells were examined. Regarding the amino acid profile changes, alanine, serine, aspartate, glutamic acid (Glu), homocysteine (HCY), and cysteine (Cysta) in the transgenic strain significantly increased, while phenylalanine (Phe), leucine (Leu), isoleucine, valine, proline (Pro), threonine (Thr), glycine (Gly), lysine, methionine (Met), and cysteine (Cys) significantly decreased (Figure 1). The metabolic flux tracing techniques were used to analyze the relevant metabolites of the TCA cycle to understand the metabolic changes in *E. coli* after the PQQ genes were transferred. The citric acid (Cit), alpha-ketoglutaric acid (α-KG), and fumaric acid (Fum) significantly increased; lactic acid (Lac) and succinic acid (Suc) showed an increasing trend; and pyruvic acid (Pyr) and malic acid significantly decreased (Figure S4a,b). Furthermore, glucose was labeled with carbon-13 isotope (13C) as a tracer to investigate the flow of carbon sources. In the protein hydrolysate of the transgenic strain, alanine significantly increased, while serine and aspartic acid significantly decreased. To a certain extent, the amino acid and TCA cycle-related metabolites of *E. coli* JM109 were affected after transformation with the PQQ genes. Therefore, PQQ may be involved in energy-metabolism-related pathways inside the cell and may even affect amino acid synthesis, allowing *E. coli* to promote growth after PQQ gene transformation.

### 3.2. Expression and Plasmid Retention in B. subtilis

After confirming that the transfer of PQQ genes induced growth effects in microorganisms, AllBioLife Corp. was commissioned to optimize the PQQ gene sequence for recognition by Gram-positive bacteria. The optimized PQQ genes were subsequently transferred into *B. subtilis*. According to previous studies, some strains of *B. subtilis* were endophytic, including *B. subtilis* 168 strain [32,33,34]. Therefore, the transgenic stable BsuM mutant strain of *B. subtilis* 168, *B. subtilis* RM125 was chosen for the endophyte experiments [35]. The optimized PQQ gene plasmid, pET-28a-pR-PQQ, was transformed into *B. subtilis*. According to our results on *E. coli*, PQQ has promotional effects on bacterial growth (Supplemental Figure S5). To create synthetic endophytic bacteria, it is necessary to first select an endophytic strain as the carrier that can express PQQ after symbiosis with plants and enhance plant growth. Additionally, according to the research by Deng et al. (2019) [33], *B. subtilis* can potentially become a plant endophyte. Thus, *B. subtilis* RM125 was selected as the transformation strain.

**Figure 2:**
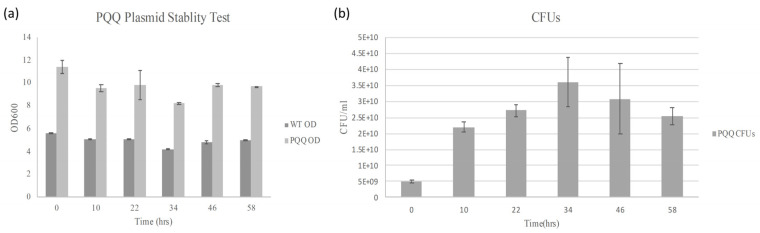
The *B. subtilis* RM125 strain carrying the PQQ gene maintained a higher growth efficiency after 58 h of antibiotic-free culturing. (**a**) The OD values at stationary phases from sampling the antibiotic-free culture within 58 h. (**b**) CFU counting on antibiotic plates within 58 h. The data are presented as the mean and standard deviation of three replicates.

It is well known that to ensure transformants retain the plasmid, the culture medium must contain antibiotics. However, to avoid potential negative effects of antibiotics in the subsequent plant experiments, it was necessary to verify whether the transformants could stably retain the plasmid and maintain its function in the absence of antibiotics after pre-culture with antibiotics. Based on the significant growth differences between the transformants and the control group (Figure 2a), the *B. subtilis* RM125 PQQ was cultured in LB without antibiotics for 58 h after pre-culture in LB with kanamycin. Within this period, *B. subtilis* samples were pulled out and cultured in another LB without antibiotics for 12 h, which was around the stationary phase of growth curves (Supplemental Figure S5), and the OD values were measured (Figure 2a). The growth comparison showed that the stationary phase OD value of *B. subtilis* RM125 containing the PQQ gene cluster was approximately double that of the control group (Supplemental Figure S5). Given this result, if the antibiotic is removed (Figure 2a), the PQQ gene could be still functional in the cells, resulting in higher growth efficiency compared with the control group, even after 58 h. Furthermore, Figure 2b shows that based on colony-forming units (CFUs) on antibiotic plates, the *B. subtilis* bacteria could retain the plasmids even without antibiotic addition during batch culturing. The results indicate potential economic value without the need for antibiotics.

### 3.3. PQQ Detection

**Figure 3:**
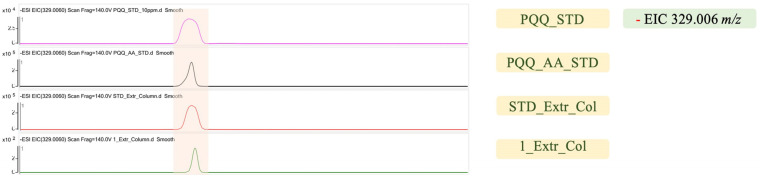
PQQ determination using LC-QTOF. The shaded area represents the same peak of compound. PQQ_STD represents PQQ standard solution; PQQ_AA_STD represents PQQ standard solution with additional amino acids; STD_Extr_Col represents the sample solution after column purification from PQQ standard solution; 1_Extr_Col represents the column purification from the culture medium of *B. subtilis* RM125 PQQ.

Although PQQ is involved in several intracellular bioprocesses [11], it could also be detectable in the culture medium [10]. 1_Extr_Col (Figure 3) represents a fermentation broth sample of *B. subtilis* RM125 PQQ which containing PQQ after organic solvent extraction and column purification, as described in Section 2. The peak appears at the same position as the standard sample containing PQQ, and it can thus be inferred that *B. subtilis* RM125 with PQQ biosynthetic genes may secrete PQQ into the extracellular environment. In addition, PQQ_AA_STD represents a PQQ standard containing amino acids. The amino acids in the samples interfere with PQQ detection; earlier studies reported similar results [36]. Under the same conditions, the greater the variety of amino acids in the solution, the lower the detectable amount of PQQ; as shown in Figure 3, the signal strength of PQQ_AA_STD was lower than that of PQQ_STD. Different amino acids also exhibit varying degrees of interference with PQQ detection, as shown in Supplemental Figure S6. Amino acids that are less likely to interfere with detection include alanine, valine, leucine, isoleucine, glutamic acid, methionine, phenylalanine, glycine, serine, lysine, and tyrosine (Supplemental Figure S6).

### 3.4. The Effect of Transformation on the Free Amino Acid Content in the Fermentation Broth

After confirming the secretion of PQQ, the free amino acids, amino acid production, and metabolic flux were measured. The free amino acid results are shown in Table 1. Compared with the *B. subtilis* RM125 host, only the L-threonine, L-serine, L-alanine, L(-)-cystine, L-methionine, L-tyrosine, L-phenylalanine, L-lysine, L-histidine, L-arginine, and tryptophan contents decreased in the *B. subtilis* RM125 PQQ strain. However, compared with LB, the L-valine, and L-proline contents increased in the *B. subtilis* RM125 PQQ strain.

### 3.5. TCA Cycle Metabolic Fluxes in B. subtilis RM125

**Figure 4:**
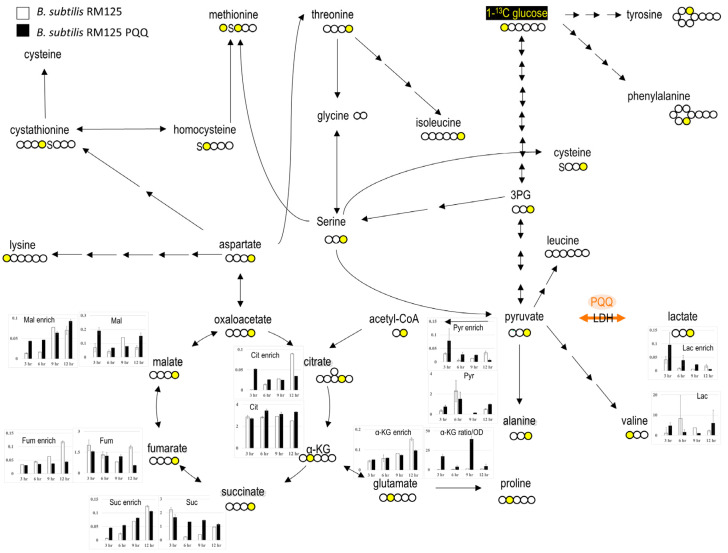
PQQ pathway expression improved TCA cycle metabolic fluxes in *B. subtilis* RM125. The *B. subtilis* RM125 strain carrying the PQQ biosynthetic genes exhibited higher levels of lactate, pyruvate, citrate, malate, and alpha-KG. The labeled carbon is marked in yellow. The lactate dehydrogenase (LDH) that PQQ may act as cofactor is highlighted. The data are presented as the mean and standard deviation of two replicates.

The *B. subtilis* strain with PQQ biosynthetic genes exhibited a growth trend similar to that of *E. coli*, with the OD value in the stable phase approaching twice that of the control group (Figure 2a). The changes in carbon flow were also tracked using glucose labeled with C^13^, as shown in Figure 4 (for high-resolution images, see Figure S4a,b). TCA cycle metabolites, succinate, and citrate significantly increased after 3 and 6 h of culturing and significantly decreased after 12 h. Compared with the RM125 wild-type strain (*n* = 2), in the 3 h culture, the isotopic enrichment of TCA cycle metabolites Suc+1, Mal+1, and Cit+1 in the RM125 PQQ strain (*n* = 2) increased by 524% (*p* < 0.001), 221% (*p* = 0.003), and 5042% (*p* < 0.001), respectively, while Fum+1 decreased by 7% (*p* = 0.004). After 6 h of incubation, the isotopic enrichment of TCA cycle metabolites Suc+1, Mal+1, and Cit+1 increased by 122% (*p* = 0.011), 180% (*p* = 0.0001), and 86% (*p* = 0.021), respectively, while Fum+1 showed a slight decreasing trend at 21% (*p* = 0.059). Like with *E. coli*, Cit, α-KG, and Suc showed varying degrees of increase, while Pyr, Mal, and Fum showed the opposite trend (Figure 4 and Figure S7a,b). Therefore, it appears that the PQQ synthesis gene has different effects on different species.

### 3.6. The Effect of B. subtilis Containing PQQ Biosynthetic Genes on the Growth of Arabidopsis thaliana

**Figure 5:**
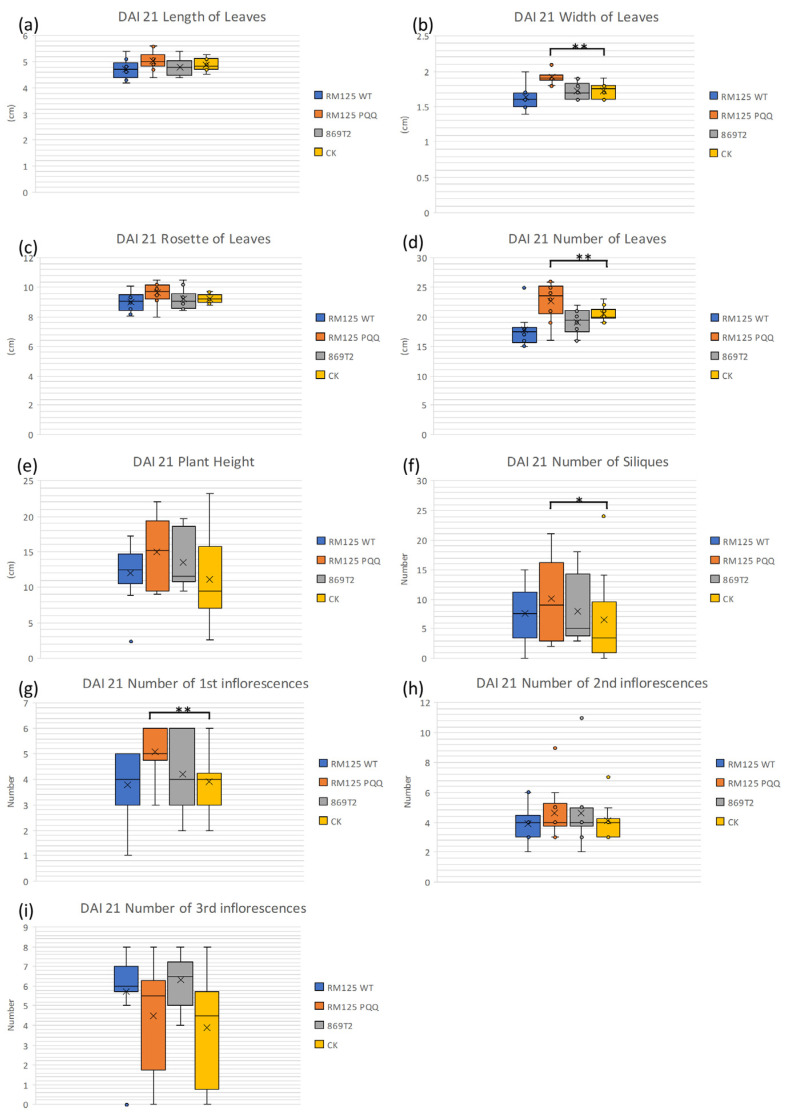
The phenotype of *Arabidopsis* was assessed after inoculation with different bacterial strains. RM125 WT, RM125 PQQ, 869T2, and CK represent the plants inoculated with *B. subtilis* RM125 WT strain, *B. subtilis* RM125 PQQ strain, *B. seminalis* 869T2 strain, and water, respectively. The RM125 PQQ strain increased the median values for (**a**) leaf length, (**b**) leaf width, (**c**) leaf rosette diameter, (**d**) number of leaves, (**e**) plant height, (**f**) siliques, (**g**) number of first inflorescences, (**h**) number of second inflorescences, and (**i**) number of third inflorescences, compared to the control group (CK) (*n* = 10, statistical analysis was performed using Student’s *t*-test; * *p* < 0.05, ** *p* < 0.01).

**Figure 6:**
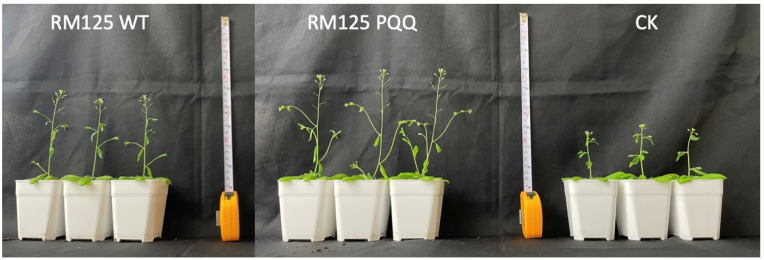
Comparison of the *Arabidopsis* phenotype between the treatment and control groups. Compared with the control group, *A. thaliana* inoculated with *B. subtilis* RM125 grew better, and RM125 with PQQ biosynthetic genes best promoted the growth of *A. thaliana*.

The impact of *B. subtilis* containing PQQ biosynthetic genes on the growth of *Arabidopsis thaliana* was investigated. According to previous findings, the PQQ-secreting strain *B. seminalis* 869T2 can provide bananas with systemic tolerance to *Fusarium oxysporum* and enhance their growth efficiency [37,38]. Therefore, the specific benefits that PQQ synthesis strains offer to plants are interesting. The above results indicated that *B. subtilis* containing the PQQ biosynthetic plasmid can stably express these genes in antibiotic-free media and thus has a higher growth efficiency than the control group (Figure 2). The *A. thaliana* was selected as the subject for subsequent experiments to test whether PQQ-producing *Bacillus* can enhance plant growth. Subsequently, the observations were made, and various measurements of *Arabidopsis* were taken on DAI 21 (21 days after inoculation), as shown in Figure 5. The CK and 869T2 groups were the negative and positive controls, respectively. It is interesting to know that the laboratory *Bacillus* host strain could actually act as an endophyte and promote plant growth when it was compared with no bacterial treatment of *A. thaliana.* On the other hand, PQQ-producing *B. subtilis* was found to promote the growth of *Arabidopsis*, with significant differences (*p* < 0.05) in leaf width, leaf number, siliques, and primary stem number compared with the control group (Figure 5). Although there were no significant differences in leaf length, leaf rosettes, plant height, or second and third inflorescence numbers, *Arabidopsis* growth efficiency improved after irrigation with *B. subtilis* containing the PQQ biosynthetic genes (Figure 5). The *Arabidopsis* phenotype on DAI 21 is shown in Figure 6, and at the same scale, *Arabidopsis* treated with transgenic *B. subtilis* grew taller and had more siliques than the control group (CK), indicating that under the same conditions, PQQ-producing *B. subtilis* promotes *Arabidopsis* growth.

## 4. Discussion

PQQ is currently considered a coenzyme, but it possesses properties similar to those of vitamins, which once led it to be considered a new kind of vitamin [39]. In addition to acting as a coenzyme in cells, assisting certain dehydrogenases in reactions, it also possesses antioxidant properties that help eliminate free radicals [40]. Interestingly, when PQQ serves as a cofactor for lactate dehydrogenase (LDH), it elevates NAD^+^ and pyruvate, further influencing the expression of sirtuin 1, which is crucial for cellular redox balance and metabolism [41]. Currently, there is only limited research on the impact of PQQ on microbial metabolism. However, through this study, preliminary insights into possible mechanisms were gained, helping us to understand more about both microbial metabolism and the effects of metabolites on plants, an area of interest for us.

Hur et al. (2024) [42] reported that microbial metabolites can either promote or inhibit plant growth. If these metabolites possess specific biosynthetic pathways, genetic engineering could be employed to introduce target genes into engineered endophytic bacteria, enabling experiments to confirm the impact of each metabolite on various plant species. These results suggest that using engineered endophytes may be a novel research strategy method for investigating the effects of specific substances on plants.

In this study, genetic engineering techniques were employed to introduce PQQ gene cluster expression in *E. coli* and *B. subtilis* RM125, with the latter having the potential to become an endophyte [43]. The pR promoter can be functional both in *E. coli* JM109 DE3 and *B. subtilis* RM125, making it an ideal promotor. Remarkably, the optimized PQQ synthesis gene cluster also promoted the growth of *B. subtilis* RM125. In this research, the expression of heterologous PQQ synthesis genes in the *B. subtilis* strain enhanced the growth of both bacteria and inoculated plants (Figures S3 and S5, Figure 2a and Figure 6). Moreover, the presence of PQQ synthesis genes altered the amino acid metabolism of bacteria. Consequently, our results demonstrate that the growth rate was enhanced through multiple metabolic pathways inside the bacterial host cells. Considering previous studies highlighting PQQ as a growth stimulant, we propose that *Bacillus* with exogenous PQQ synthesis genes acts as an engineered endophyte, aiding plant growth. Metabolic changes within the cells were observed, using various metabolites from the TCA cycle as examples. Interestingly, PQQ is involved in certain dehydrogenase reactions, such as lactate dehydrogenase, enabling lactate dehydrogenase to convert lactate into pyruvate [11], increasing the pyruvate concentration, and directly affecting the metabolite concentration of the TCA cycle. Although there is no research indicating what metabolic impact PQQ may have on microorganisms, this may be one of the reasons for the different end-product concentrations observed in different bacterial species. The outcomes may exhibit varying levels of plant growth-promoting effects. For instance, while *Rhodopseudomonas palustris* possesses PQQ synthesis genes and can produce PQQ under different culture conditions [44], certain strains may still be ineffective [45].

In the plant growth process, microorganisms and nutrients play crucial roles. Particularly during the early stages of growth, appropriately inoculating specific microbial strains enhances plant disease resistance and growth efficiency [32]. Genetically engineering microorganisms to secrete specific substances enables a more direct verification of the impact of these substances on plants. *B. subtilis* RM125 was used to express the PQQ genes, and a bacterial pellet solution was applied during irrigation to stimulate plant growth (Figure 5 and Figure 6). In addition to assessing plant growth, metabolic changes in *B. subtilis* RM125 were also analyzed. This suggests that the *B. subtilis* PQQ strain can produce proline. Proline is believed to help plants survive high temperatures and drought stress [46], meaning that, in addition to the direct effects of PQQ, the metabolic composition of the transgenic strain changes due to PQQ, for example, by increasing the proline content in the fermentation broth. Other metabolites might also provide additional nutrients to the plants, leading to growth-promoting effects. However, the underlying mechanism of this action requires further experimental verification. The growth-promoting effects of PQQ on both microorganisms and plants were demonstrated. Additionally, the microbial expression of specific gene clusters was utilized to assess whether the target substance could influence plant growth. The results of this study provide a platform for examining the potential impact of specific substances on plants.

As mentioned in the introduction, plant endophytes can compete for nutrients and space in the roots with pathogens [5]. According to a previous bioinformatics analysis, growth rate was identified as a key factor in dominating the rhizosphere [47]. Upon the expression of the PQQ genes, both *E. coli* and *B. subtilis* strains exhibited nearly double the growth efficiency (Figure S3 and Figure 2a). In addition to the plant growth-promoting effects observed in the *B. subtilis* RM125 wild-type strain (Figure 6), the *B. subtilis* RM125 PQQ strain, which exhibited a higher growth rate, could be considered a superior plant growth-promoting microorganism.

## 5. Conclusions

This study was aimed at investigating the impact of PQQ on microbial metabolism and plant growth. The results indicated that PQQ influenced the composition of certain microbial metabolites, which, in turn, appeared to have positive effects on plant growth. Additionally, there are many questions that remain unanswered, such as how many of the microbial metabolites involved in plant growth are actually affected. Nevertheless, a platform has been established that can be used to evaluate the effects of specific substances on plant growth.

## Figures and Tables

**Table 1:** Free amino acid detection in the fermentation broth.

Amino Acid	Samples			Differences Between	
	LB (mg/100 g)	*B. subtilis* RM125 (mg/100 g)	*B. subtilis* RM125 PQQ (mg/100 g)	*B. subtilis* RM125 PQQ and RM125 Strains (mg/100 g)	*B. subtilis* RM125 PQQ and LB (mg/100 g)
L-Aspartic Acid	9.04 ± 0.04	0.38 ± 0.02	1.37 ± 0.01	0.99	−7.66
L-Threonine	16.95 ± 0.07	9.17 ± 0.74	0.57 ± 0.06	−8.59	−16.37
L-Serine	18.36 ± 0.07	0.63 ± 0.03	0.41 ± 0.04	−0.22	−17.94
L-Glutamic acid	40.62 ± 0.15	2.20 ± 0.06	3.90 ± 0.10	1.7	−36.72
Glycine	7.41 ± 0.03	1.01 ± 0.17	3.77 ± 6.26	2.76	−3.63
L-Alanine	31.61 ± 0.12	0.95 ± 0.04	0.34 ± 0.08	−0.61	−31.27
L-Valine	26.92 ± 0.11	24.59 ± 0.48	31.02 ± 0.11	6.42	4.09
L(-)-Cystine	2.67 ± 0.01	1.95 ± 0.02	1.15 ± 0.01	−0.79	−1.51
L-Methionine	10.58 ± 0.02	11.45 ± 0.02	7.81 ± 0.09	−3.64	−2.77
L-Isoleucine	21.85 ± 0.09	13.27 ± 0.60	19.21 ± 0.12	5.94	−2.64
L-Leucine	66.41 ± 0.22	44.76 ± 2.11	50.72 ± 0.44	5.96	−15.68
L-Tyrosine	7.94 ± 0.02	8.36 ± 0.04	7.21 ± 0.02	−1.14	−0.73
L-Phenylalanine	37.19 ± 0.18	35.04 ± 0.20	31.52 ± 0.09	−3.51	−5.67
Tryptophan	11.87 ± 0.02	11.61 ± 0.11	11.50 ± 0.08	−0.10	−0.36
L-Lysine	60.09 ± 0.22	62.94 ± 0.31	55.30 ± 0.10	−7.64	−4.79
L-Histidine	5.54 ± 0.01	4.84 ± 0.63	0.24 ± 0.27	−4.59	−5.3
L-Arginine	36.87 ± 0.13	0.53 ± 0.02	0.00 ± 0.00	−0.52	−36.87
L(-)-Proline	6.16 ± 0.03	0.00 ± 0.00	6.86 ± 0.35	6.86	0.7

## Data Availability

The raw data supporting the conclusions of this article will be made available by the authors on request due to the raw data of GC-MS analysis were too many to show in a scientific article.

## Supplementary Materials

The following supporting information can be downloaded at: https://www.mdpi.com/article/10.3390/microorganisms13020293/s1, Figure S1: PQQ gene cluster; Figure S2: The amino acid fluxes of *E. coli* JM109 with pET-28a or pET-28a-PQQ; Figure S3: *E. coli* growth curves; Figure S4: The tricarboxylic acid (TCA) fluxes and TCA metabolite levels in *E. coli* JM109 with pET-28a or pET-28a-PQQ; Figure S5: *B. subtilis* growth curves; Figure S6: Different amino acids exhibit varying degrees of interference with PQQ detection; Figure S7: pET-28a-pR-PQQ improved TCA metabolic fluxes in *B. subtilis* RM125; Figure S8: Plasmid map of pET-28a-T7-PQQ; Figure S9: Plasmid map of pET-28a-pR-PQQ.
