# Supplementary material for: Development of an Engineered Bacterial Endophyte: Promoting Plant Growth Through Pyrroloquinoline Quinone (PQQ) Synthesis
Source: Microorganisms. 2025 Jan 28;13(2):293. doi: 10.3390/microorganisms13020293 (PMC11858353; doi:10.3390/microorganisms13020293)
Supplement: Supplementary file 1 [file microorganisms-13-00293-s001.zip › microorganisms-3421543-supplementary.pdf]

Supplementary Material of

Development of an Engineered Bacterial Endophyte: Promoting Plant Growth Through Pyrroloquinoline Quinone (PQQ) Synthesis

Ti Fang [1,2,3], Shou-Chen Lo [1], Yu-Ning Yu [1], Nga-Lai Sou [4,5,6], Shih-Hsun Walter Hung [1,7], Jian-Hau Peng [4,5,6,8,9], En-Pei Isabel Chiang [4,5,6,8,9] and Chieh-Chen Huang [1,5,6,8,9,*]

1 Department of Life Science, National Chung Hsing University, Taichung 402, Taiwan; quinnfang@hotmail.com (T.F.); scl@dragon.nchu.edu.tw (S.-C.L.); aoy92106@gmail.com (Y.-N.Y.); walter030170@gmail.com (S.-H.W.H.)

2 Biotechnology Program of Industry-Academia Collaboration, National Chung Hsing University, Academia Sinica, Taichung 402, Taiwan

3 Biotechnology Program of Industry-Academia Collaboration, Academia Sinica, Taipei 115, Taiwan

3 Department of Food Science and Biotechnology, National Chung Hsing University, Taichung 402, Taiwan; looksusan2013@gmail.com (N.-L.S.); jianhau.peng@gmail.com (J.-H.P.); chiangisabel@nchu.edu.tw (E.-P.I.C.)

4 Innovation and Development Center of Sustainable Agriculture (IDCSA), National Chung Hsing University, Taichung 402, Taiwan

5 Advanced Plant and Food Crop Biotechnology Center (APFCBC), National Chung Hsing University, Taichung 402, Taiwan

6 Institute of Plant and Microbial Biology, Academia Sinica, Taipei 115, Taiwan

8 Doctoral Program in Microbial Genomics, National Chung Hsing University, Taichung 402, Taiwan

9 Doctoral Program in Microbial Genomics, Academia Sinica, Taipei 115, Taiwan

* Correspondence: cchuang@dragon.nchu.edu.tw; Tel.: +886-04-22840416 (ext. 405)

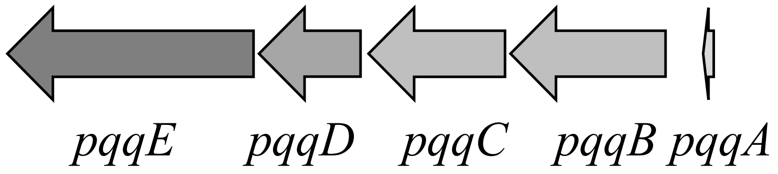

Figure S1. PQQ gene cluster. The plasmid construct containing the PQQ gene cluster is based on *Gluconobacter oxydans*, as its PQQ gene cluster has relatively shorter base pairs and fewer genes, which can reduce the synthesis cost. pqqA is responsible for carrying tyrosine and glutamine, usually with an interval of three amino acids in between, to facilitate folding by PqqB, bringing tyrosine closer to glutamine. Subsequently, binding is performed by PqqD, PqqE, and PqqF. In *E. coli*, TldD executes the function of PqqF. After binding is completed, a protease removes the excess portions, and finally, oxidation to generate the third ring structure modification is carried out by PqqC.

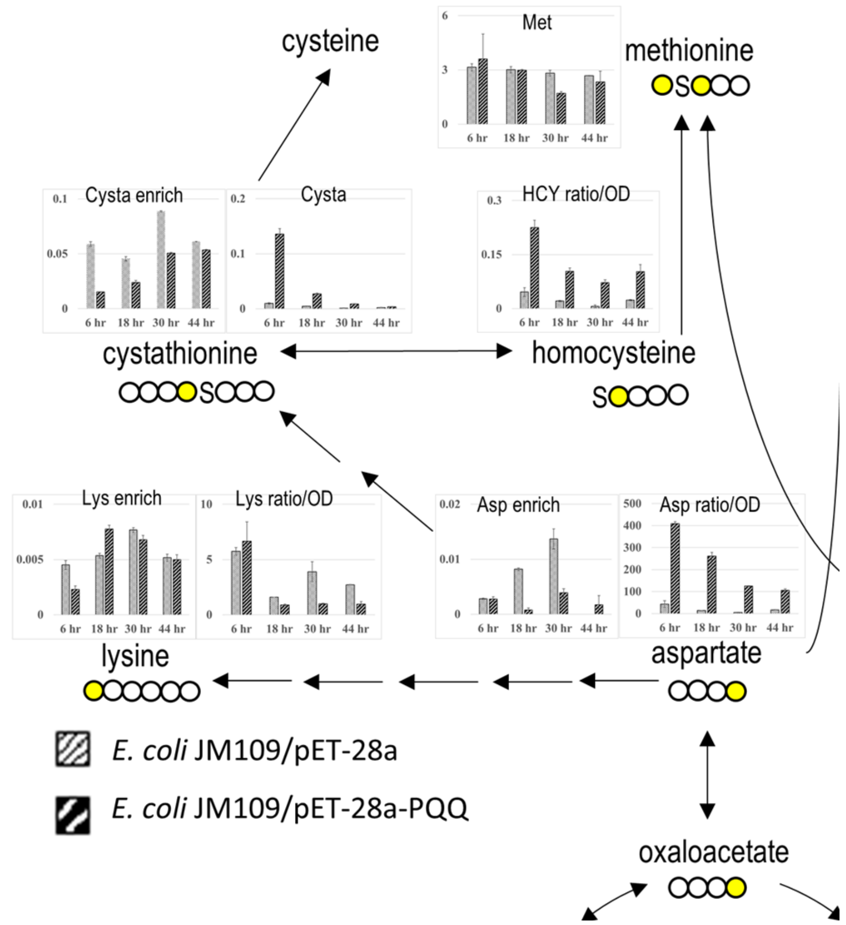

Figure S2a. The amino acid fluxes of *E. coli* JM109 with pET-28a or pET-28a-PQQ. pET-28a-PQQ improved amino acid fluxes and production in *E. coli* JM109, and yellow circle is ^{13}C. In this figure, it shows homocysteine, cystathionine and aspartate are increased.

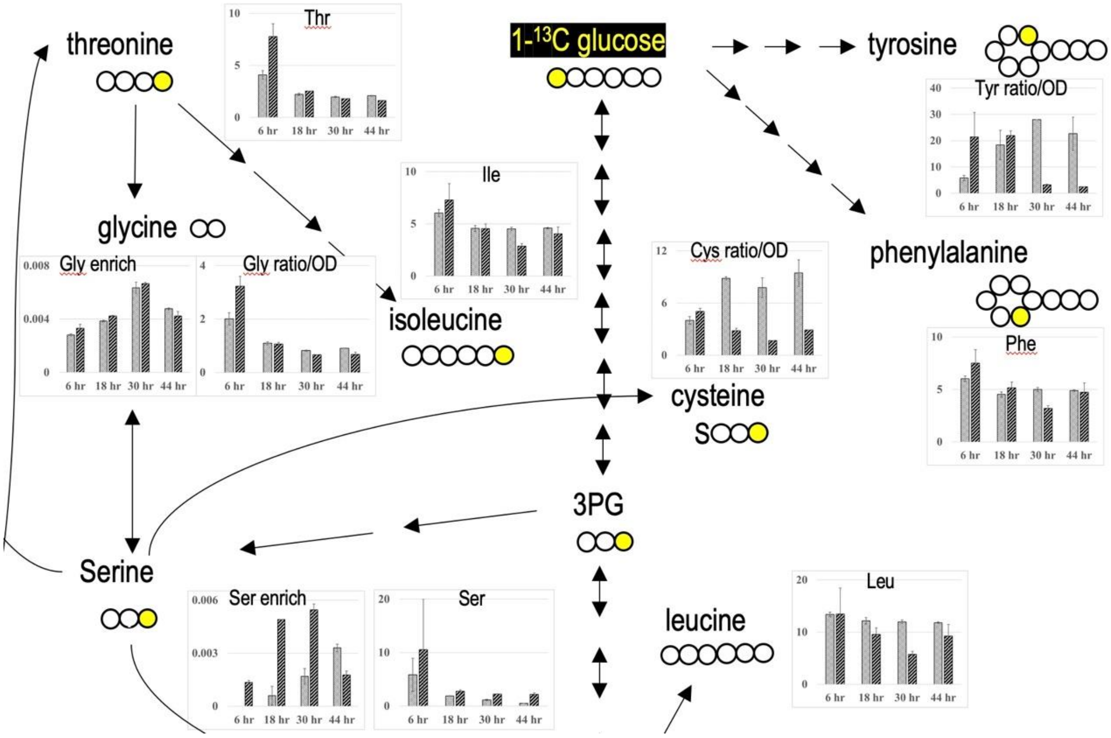

Figure S2b. The amino acid fluxes of *E. coli* JM109 with pET-28a or pET-28a-PQQ. pET-28a-PQQ improved amino acid fluxes and production in *E. coli* JM109, and yellow circle is ^{13}C. In this figure, it shows threonine, glycine and serine are increased.

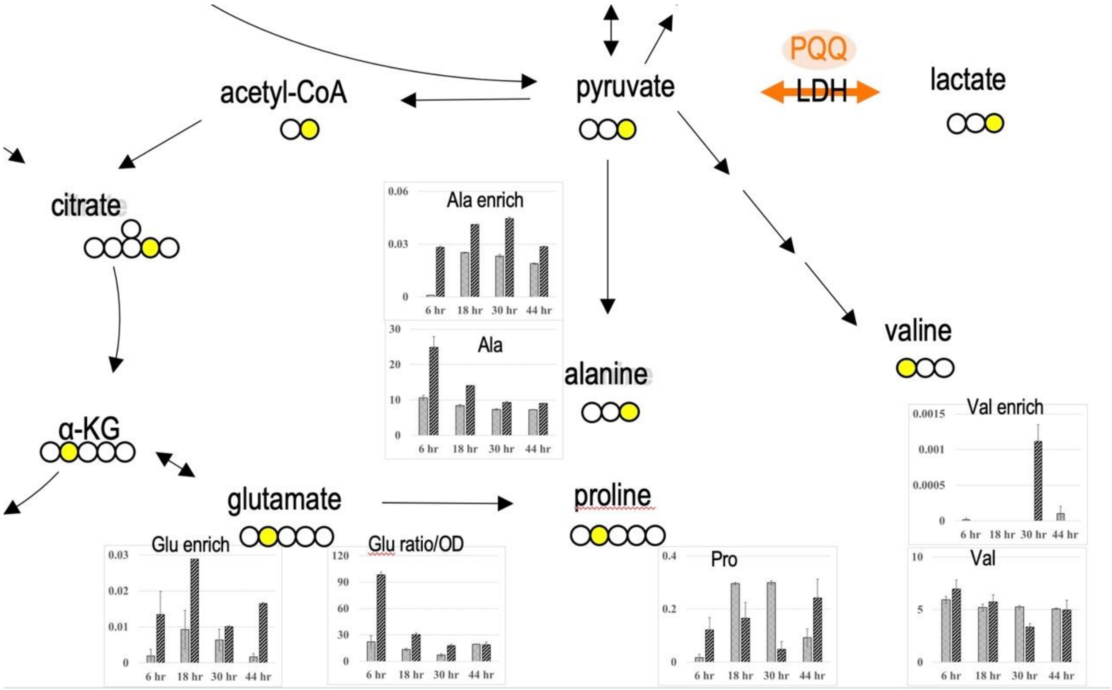

Figure S2c. The amino acid fluxes of *E. coli* JM109 with pET-28a or pET-28a-PQQ. pET-28a-PQQ improved amino acid fluxes and production in *E. coli* JM109, and yellow circle is ^{13}C. In this figure, it shows alanine, glutamate were increased.

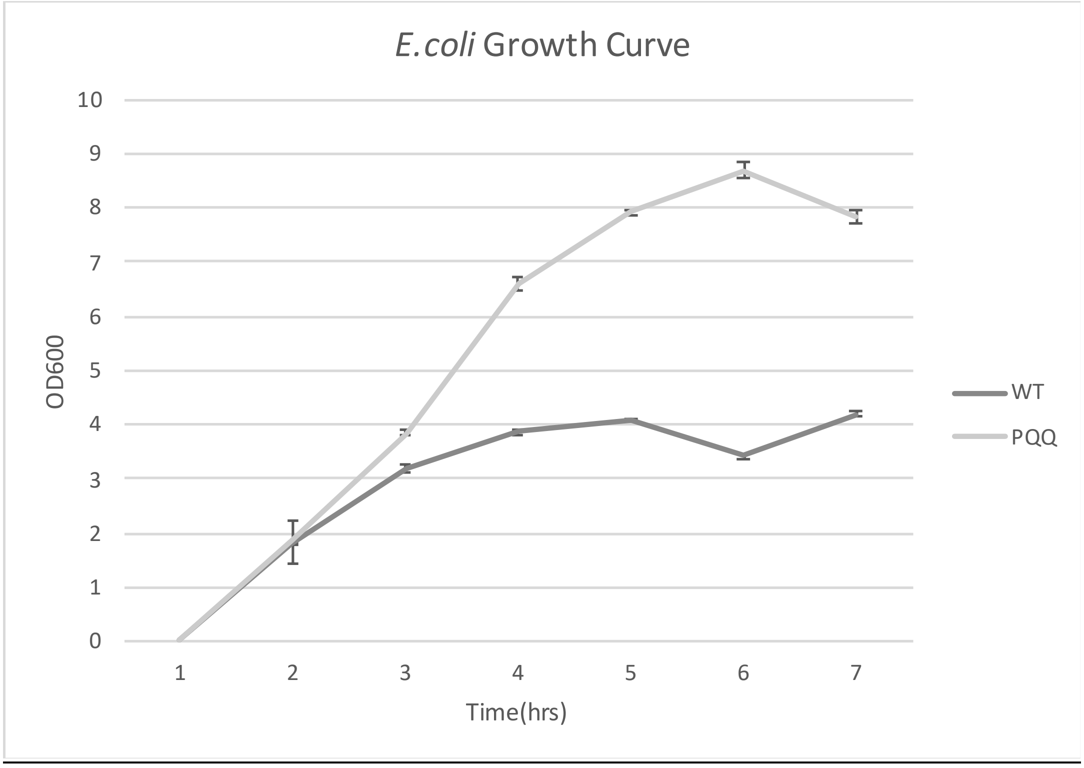

Figure S3. *E. coli* growth curves. With pET-28a-T7-PQQ achieved growth promotion, resulting in a final OD value that was approximately double that of the control group.

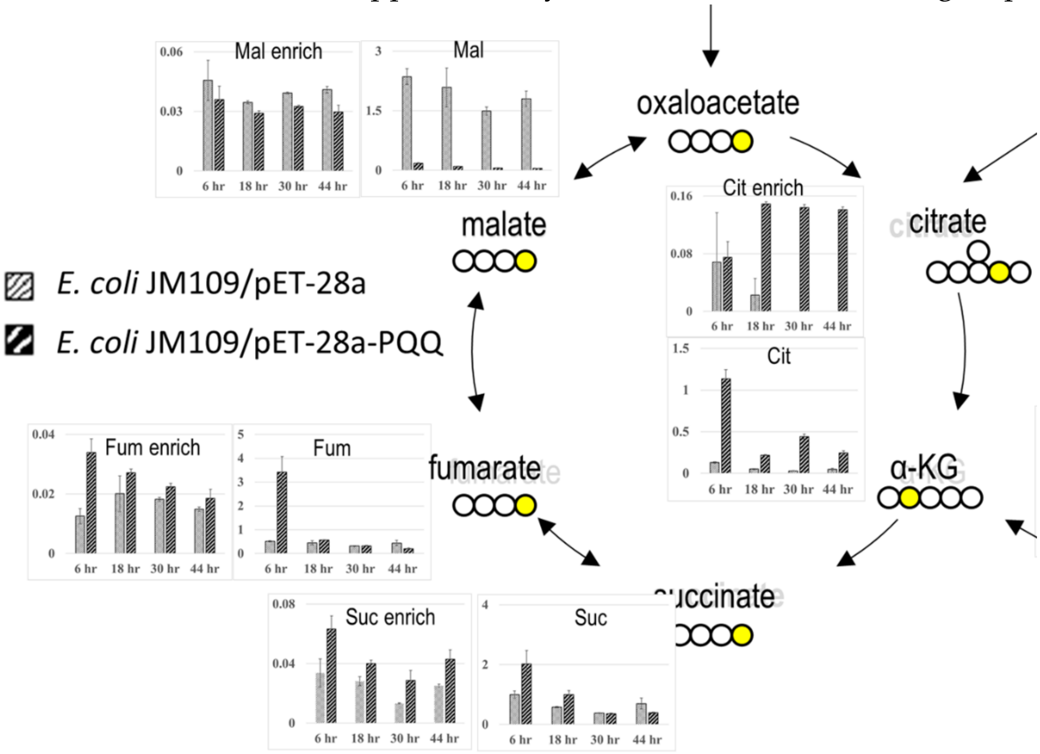

Figure S4a. The tricarboxylic acid (TCA) fluxes and TCA metabolite levels in *E. coli* JM109 with pET-28a or pET-28a-PQQ. Yellow circle is ^{13}C. And as figure shows, citrate was increased, malate was decreased, succinate was increased before 18 hours.

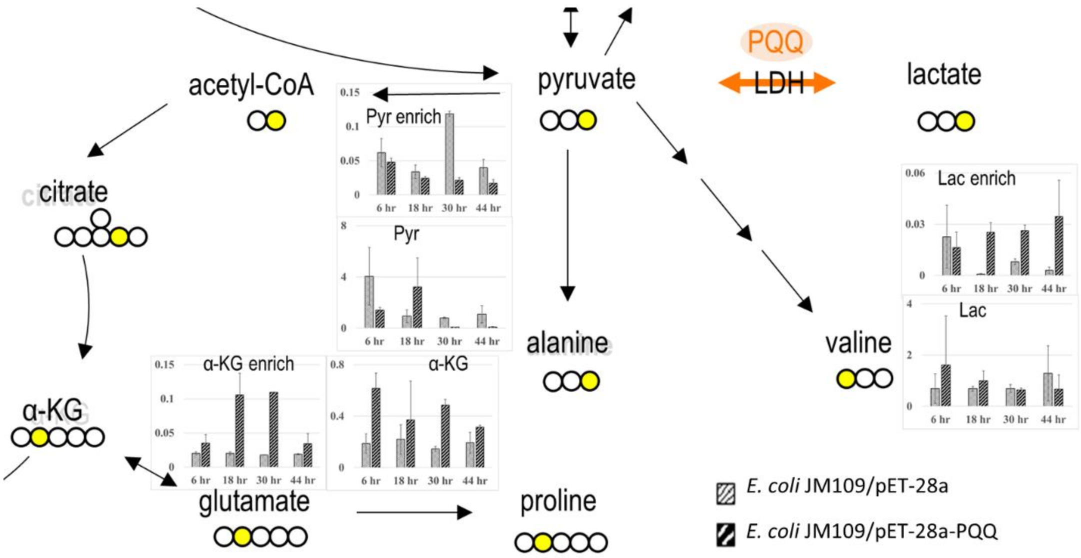

Figure S4b. The tricarboxylic acid (TCA) fluxes and TCA metabolite levels in *E. coli* JM109 with pET-28a or pET28a-PQQ. Yellow circle is ^{13}C. As figure shows, the enrichment of lactate was increased, and α-KG was also increased.

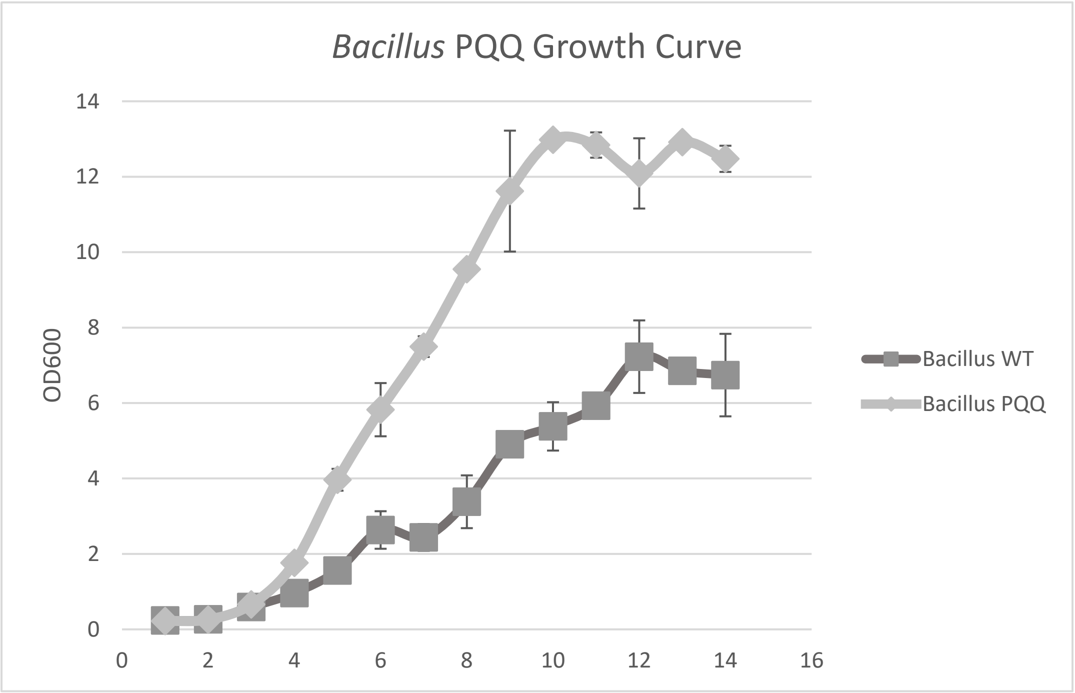

Figure S5. *B. subtilis* growth curves. With pET-28a-pR-PQQ achieved growth promotion, resulting in a final OD value that was approximately double that of the control group.

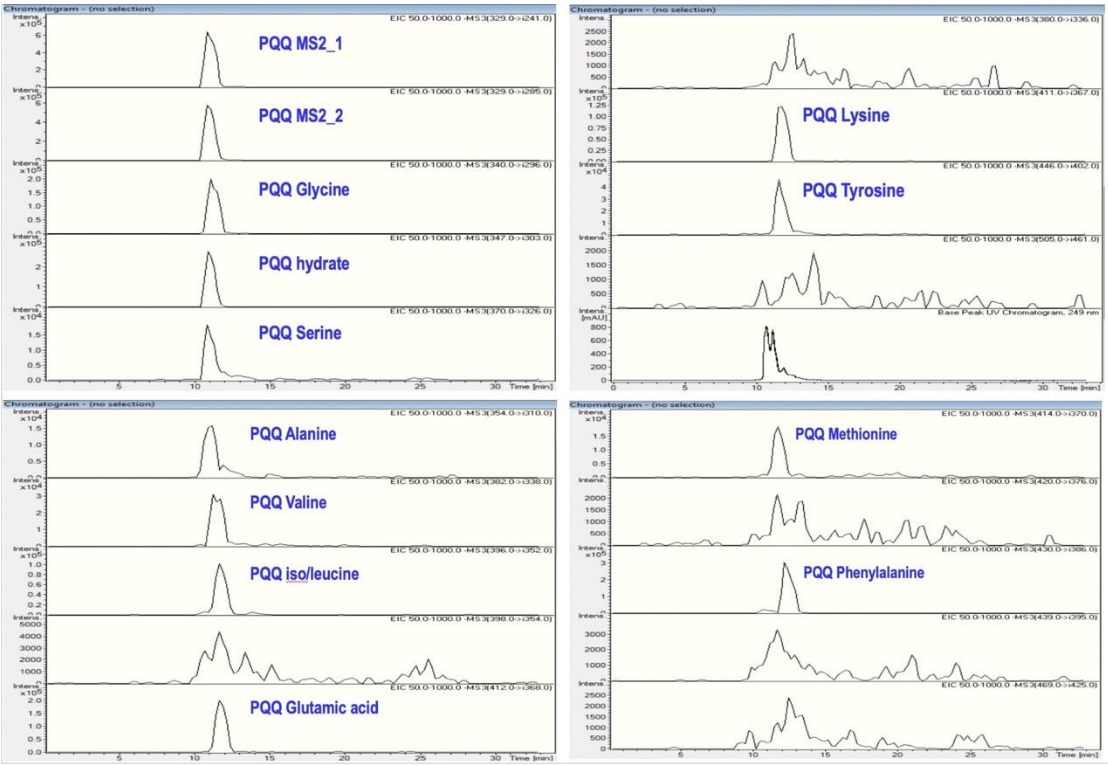

Figure S6. Different amino acids exhibit varying degrees of interference with PQQ detection. The results revealed that some amino acids exhibit less noticeable interference in the detection of PQQ. These include Alanine, Valine, Leucine, Isoleucine, Glutamic acid, Methionine, Phenylalanine, Glycine, Serine, Lysine, and Tyrosine.

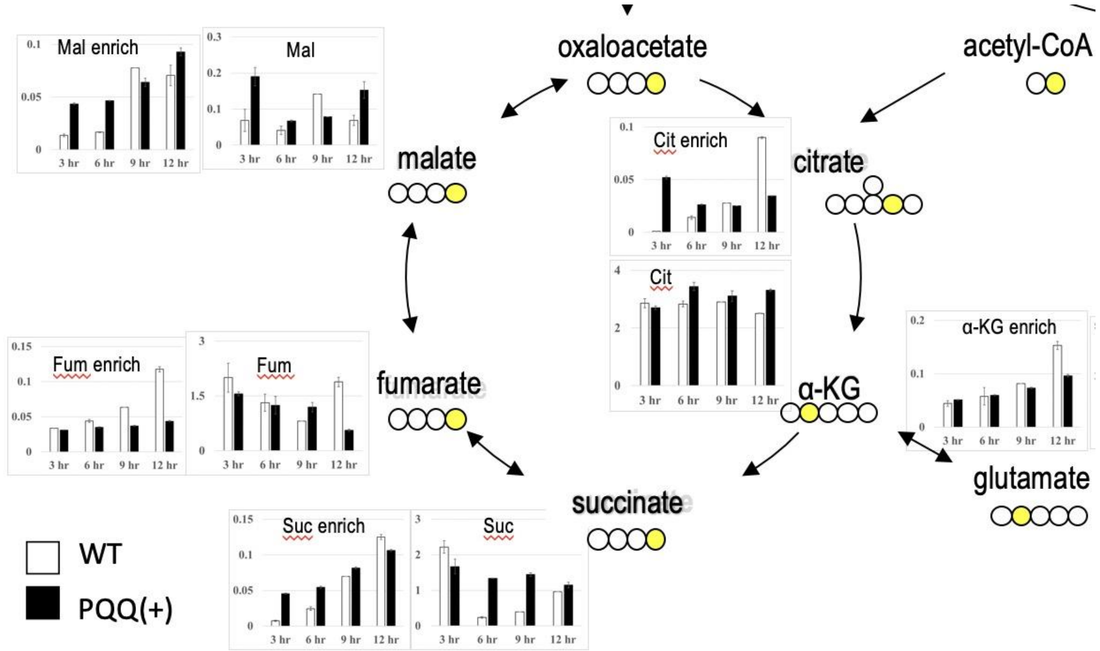

Figure S7a. **pET-28a-pR-PQQ improved TCA metabolic fluxes in *B. subtilis* RM125**. Yellow circle is ^{13}C. As figure shows, malate is increased.

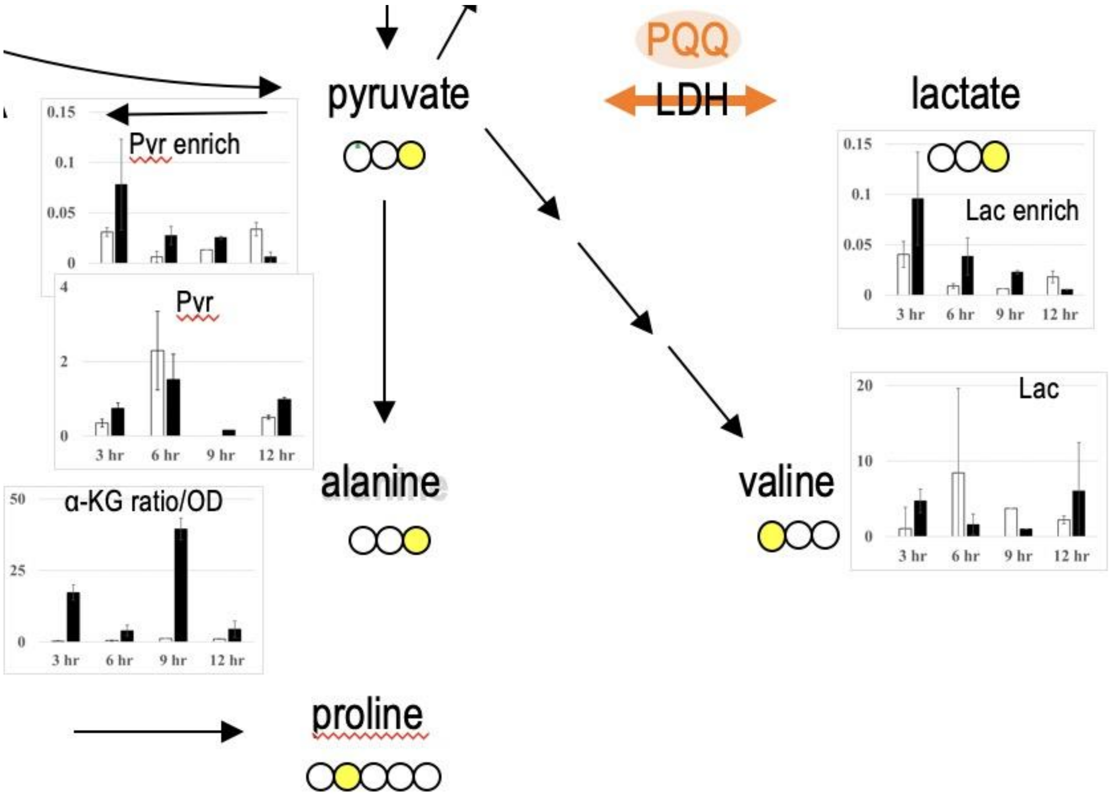

Figure S7b. pET-28a-pR-PQQ improved TCA metabolic fluxes in *B. subtilis* RM125. Yellow circle is ^{13}C. As figure shows, the enrichment of pyruvate and lactate are incresed. And α-KG is increased in per unit of OD value.

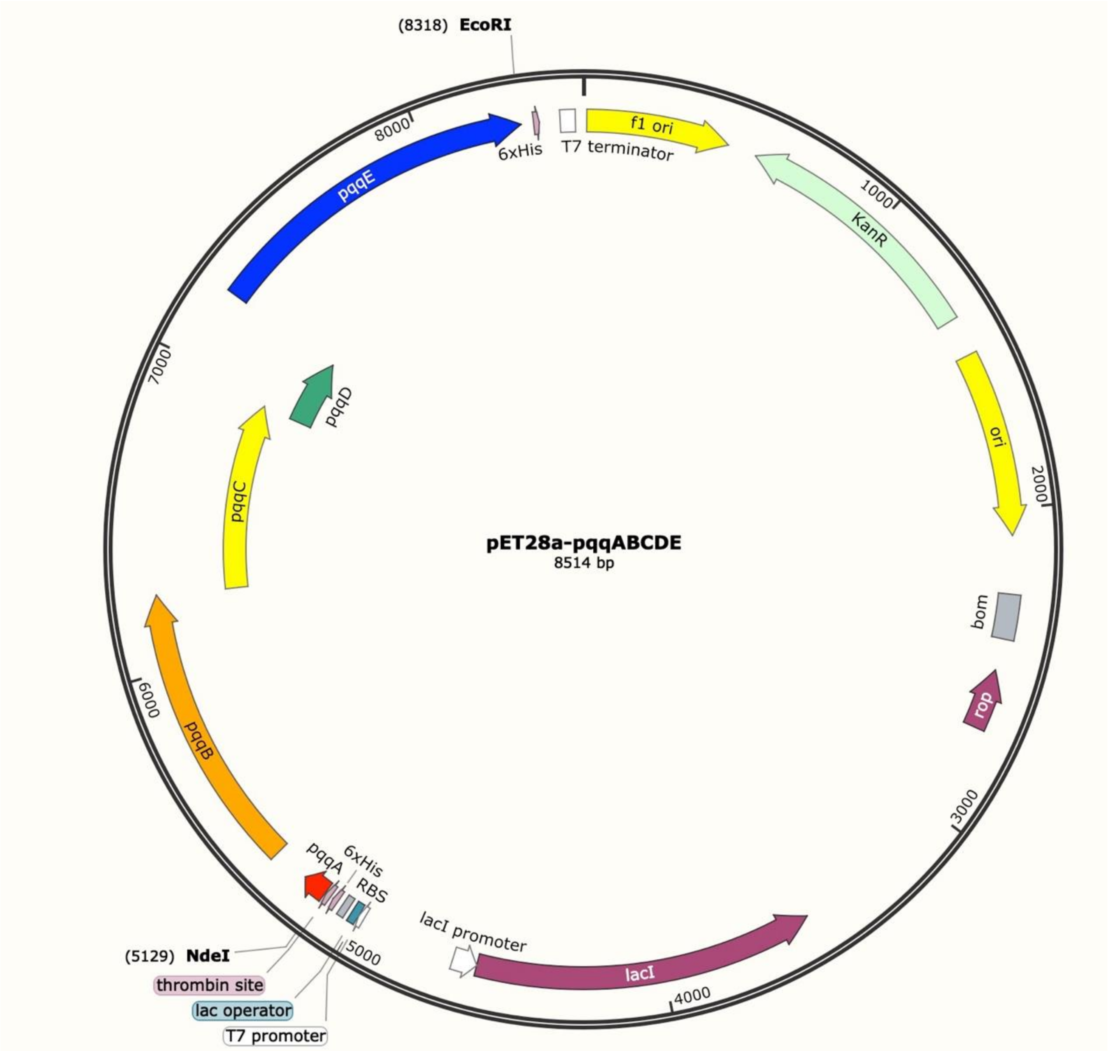

Figure S8. Plasmid map of pET-28a-T7-PQQ. The pET-28a plasmid with the T7 promoter was used to enable *E. coli* JM109 (DE3) to express the PQQ biosynthesis genes.

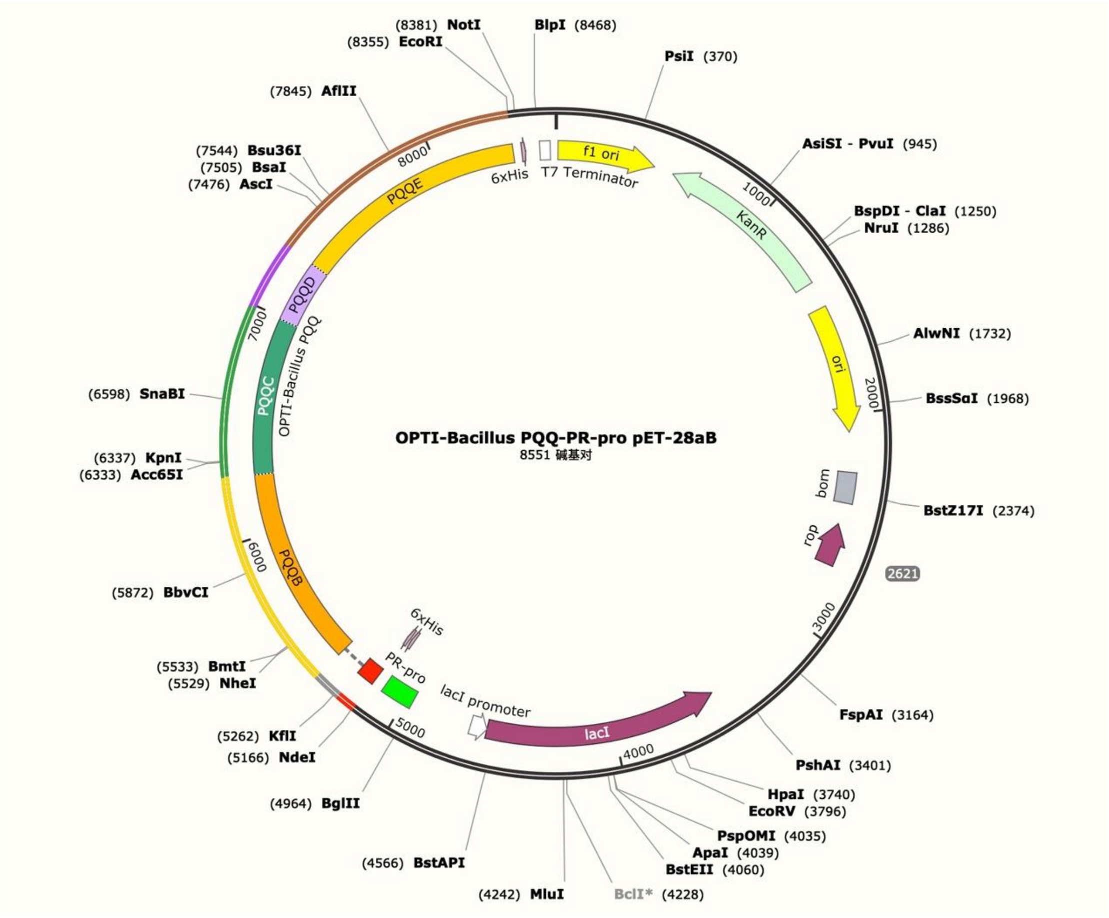

Figure S9. Plasmid map of pET-28a-pR-PQQ. The pET-28a plasmid with the pR promoter was used to enable *B. subtilis* RM125 to express the PQQ biosynthesis genes.

<u>The sequence of PQQ gene cluster in pET-28a-T7-PQQ</u>

Purple is T7 promoter, red is pqqA, orange is pqqB, brown is pqqC, green is pqqD blue is pqqE, grey is terminator. Base pair with <u>underline</u> is overlapping area.

TAATACGACTCACTATAGGGGAATTGTGAGCGGATAACAATTCCCCTCTAGAAATA
ATTTTGTTTAACTTTAAGAAGGAGATATACCATGGGCAGCAGCCATCATCATCATCAT
CACAGCAGCGGCCTGGTGCCGCGCGGCAGCCATATGGCCTGGAACACACCGAAAG
TTACCGAAATCCCGCTGGGCGCAGAAATCAACTCGTATGTCTGCGGCGAGAAGAAA
TAAGCCGCTTTCCCGGGGACCCGTCCTTGAGGAATAATGGCACGGCCGCTCCCCCAT
GGAGCGGCCGTTTTCGTTCATGGGTGCTCTGTGGTGCCCCAGTCAGACGGTTTGTGA
AAAAATGATTGATGTCATCGTGCTTGGCGCGGCGGCAGGGGGCGGTTTTCCGCAGT
GGAACTCCGCAGCACCCGGCTGTGTGGCCGCCCGCACGCGACAGGGCGCGAAAGC
CCGGACCCAGGCCTCCCTTGCCGTCAGTGCCGACGGAAAGCGCTGGTTCATTCTCA
ACGCCTCGCCCGATCTGCGGCAGCAGATCATCGATACGCCGGCCCTGCATCATCAG
GGCAGCCTGCGTGGAACGCCCATTCAGGGCGTCGTCCTGACCTGCGGCGAGATCGA
CGCCATAACCGGGCTTCTGACCCTGCGTGAGCGTGAGCCTTTTACCCTGATGGGCAG
CGACTCGACCCTTCAGCAGCTTGCGGACAATCCGATCTTCGGTGCGCTCGATCCGGA
AATCGTCCCACGTGTTCCGCTCATTCTCGATGAAGCCACGTCCCTGATGAACAAGGA
CGGGATTCCGTCCGGTCTTTTGCTCACGGCCTTCGCCGTTCCGGGCAAGGCGCCGCT
TTACGCGGAAGCCGCAGGGTCACGCCCGGACGAGACGCTGGGCCTTTCCATTACGG

ATGGATGCAAGACGATGCTCTTCATTCCCGGCTGTGCGCAGATCACGTCGGAAATCG
TGGAACGGGTAGCGGCAGCCGATCTCGTGTTCTTTGACGGGACACTGTGGCGGGAT
GACGAAATGATCCGCGCCGGGTTGAGCCCGAAGAGCGGACAGCGGATGGGACATG
TGTCCGTGAATGATGCCGGGGGACCGGTCGAATGTTTCACGACATGCGAAAAACCC
CGTAAAGTGTTGATTCATATCAACAACTCCAATCCAATTCTGTTCGAAGACAGCCCC
GAACGCAAAGACGTCGAACGCGCCGGATGGACGGTTGCGGAAGACGGCATGACTT
TCAGACTGGACACACCATGACGCTCCTCACACCTGACCAGCTTGAAGCACAGCTTC
GCCAGATCGGGGCCGAGCGGTATCACAACCGGCACCCGTTCCATCGCAAGCTGCAT
GACGGCAAGCTGGACAAGGCACAGGTTCAGGCTTGGGCGCTGAACCGCTATTATTA
TCAGGCCCGCATCCCGGCGAAGGATGCGACGCTTCTCGCACGTCTGCCGACGGCCG
AACTGCGCCGCGAATGGCGTCGCCGGATCGAGGACCATGACGGCACGGAGCCCGG
AACGGGCGGTGTTGCGCGCTGGCTGATGCTGACGGATGGTCTGGGGCTGGACCGGG
ATTATGTGGAAAGCCTCGATGGTCTGCTTCCAGCCACGCGCTTCTCGGTCGATGCCT
ATGTGAACTTCGTGCGGGACCAGTCGATTCTGGCGGCCATTGCGTCGTCGCTGACGG
AACTGTTTTCGCCCACGATCATCAGCGAGCGCGTCTCGGGGATGCTGCGGCACTACG
ACTTTGTGTCGGAAAAGACGCTGGCCTATTTCACGCCGCGCCTGACGCAGGCCCCG
CGGGATTCCGATTTCGCGCTGGCCTATGTCCGCGAAAAGGCCCGCACGCCGGAGCA
GCAGAAAGAAGTCCTGGGAGCGCTGGAGTTCAAGTGCTCCGTGCTGTGGACGATGC
TGGATGCGCTCGACTACGCCTATGTGGAAGGCCACATTCCGCCGGGGGCTTTCGTTC
CATGACGGAGGCCCCGCATGTCGTGGCGGAGGGGACGGTTCTCTCCTTTGCCCGGG
GGCATCGTCTCCAGCACGATCGTGTGCGGGACGTGTGGATCGTGCAGGCGCCTGAA
AAAGCATTTGTAGTTGAGGGCGCCGCGCCGCATATTCTGCGGCTGCTGGATGGGAA
GCGCAGCGTCGGCGAGATCATCCAGCAGCTTGCAATCGAGTTTTCCGCCCCGCGTG
AGGTCATTGCGAAAGATGTCCTCGCGCTTCTTTCTGAACTGACAGAAAAGAACGTC
CTGCACACATGACACTCCCTTCGCCGCCGATGAGCCTTCTGGCTGAACTGACGCATC
GATGCCCGCTTTCCTGCCCCTACTGCTCCAATCCGCTTGAACTCGAACGCAAGGCGG
CAGAACTCGACACGGCCACCTGGACTGCCGTACTGGAGCAGGCGGCCGAGCTTGG
GGTGCTCCAGGTTCATTTCTCTGGCGGCGAGCCTATGGCGCGGCCTGATCTGGTCGA
ACTGGTCTCCGTCGCACGGAGACTCAACCTGTATTCCAACTTGATCACGTCCGGCGT
GTTGCTGGACGAACCGAAACTGGAAGCTCTCGACAGGGCGGGGCTGGATCACATC
CAGCTCTCTTTCCAAGACGTGACGGAGGCGGGAGCCGAGCGTATCGGCGGTCTCAA
GGGAGCGCAGGCCCGCAAGGTTGCGGCGGCGCGGCTCATCCGCGCGTCCGGCATTC
CGATGACGCTCAATTTTGTGGTGCACAGGGAAAATGTCGCCCGTATCCCCGAGATGT
TCGCCCTGGCGCGGGAACTCGGAGCGGGGCGGGTGGAGATCGCGCATACCCAGTAT
TATGGCTGGGGGCTGAAAAACCGTGAGGCGCTTCTTCCCAGCCGGGATCAGCTGGA
GGAATCCACACGCGCCGTGGAAGCGGAGCGCGCTAAGGGTGGTTTGTCCGTTGATT
ATGTGACGCCGGACTATCATGCAGACCGGCCCAAGCCCTGCATGGGGGGATGGGGC
CAGCGTTTCGTGAATGTCACACCTTCGGGCCGGGTCCTGCCGTGTCATGCAGCCGAA
ATCATTCCGGATGTCGCATTCCCGAATGTGCAGGATGTGACCCTGTCCGAAATCTGG
AACATCTCACCGCTGTTCAACATGTTCCGCGGGACGGACTGGATGCCGGAGCCCTG
CCGCTCCTGCGAGCGCAAGGAGCGTGACTGGGGCGGGTGTCGCTGTCAGGCGATG
GCGCTGACGGGGAATGCCGCGAATACCGATCCCGTATGCAGTCTCTCCCCCTATCAC

GATCGGGTGGAGCAGGCCGTCGAGAACAACATGCAGCCAGAAAGCACGTTGTTCT
ACAGGCGTTATACGTAAGAATTCGAGCTCCGTCGACAAGCTTGCGGCCGCACTCGA
GCACCACCACCACCACCACTGAGATCCGGCTGCTAACAAAGCCCGAAAGGAAGC
TGAGTTGGCTGCTGCCACCGCTGAGCAATAACTAGCATAACCCCTTGGGGCCTCTAA
ACGGGTCTTGAGGGGTTTTTTG

The sequence of PQQ gene cluster in pET-28a-pR-PQQ

Purple is pR promoter, red is pqqA, orange is pqqB, brown is pqqC, green is pqqD blue is pqqE, grey is terminator. Base pair with underline is overlapping area.

AGATCTCGATCCCGCGAAATTCCTTAAATCTATCACCGCAAGGGATAAATATTTAAC
ACCGTGCGTGTTGACTATTTTACCTCTGGCGGTGATAATGGTTGCATGTACTAAGGCC
TCTAGAAATAATAAAGGAGGTGTTATACCATGGGCAGCAGCCATCATCATCATCATC
ACAGCAGCGGCCTGGTGCCGCGCGGCAGCCATATGGCGTGGAATACACCGAAAGTT
ACAGAAATTCCGCTGGGCGCGGAAATTAATAGCTATGTTTGCGGCGAGAAGAAGTA
AGCCGCTTTCCCGGGGACCCGTCCTTGAGGAATAATGGCACGGCCGCTCCCCCATG
GAGCGGCCGTTTTCGTTCATGGGTGCTCTGTGGTGCCCCAGTCAGACGGTTTGTGAA
AAAATGATTGATGTTATTGTTCTGGGCGCGGCGGCGGGCGGCGGCTTTCCGCAATGG
AACTCAGCGGCGCCGGGCTGCGTTGCGGCGAGAACAAGACAAGGCGCGAAAGCG
AGAACACAAGCAAGCCTCGCCGTCAGTGCCGACGGAAAGAGATGGTTCATACTAA
ACGCTAGCCCAGACCTACGACAGCAGATAATAGACACACCGGCGCTGCATCATCAA
GGCTCACTGAGAGGCACACCGATTCAAGGCGTTGTTCTGACATGCGGCGAAATTGA
TGCCATCACCGGATTACTTACTTTGCGGGAGCGTGAGCCATTCACCCTTATGGGCTC
AGATTCAACACTGCAACAACTGGCGGATAATCCGATATTCGGAGCACTTGATCCGG
AAATTGTTCCGAGAGTTCCGCTGATTCTGGATGAAGCGACATCACTGATGAATAAAG
ATGGCATTCCGTCAGGCCTGCTGCTGACAGCGTTTGCGGTTCCAGGGAAGGCACCC
TTATACGCTGAGGCAGCAGGTTCTCGTCCTGACGAAACGCTAGGATTGTCAATTACT
GATGGCTGTAAAACGATGCTTTTCATTCCAGGTTGTGCCCAGATAACTTCCGAGATC
GTGGAGCGTGTGGCGGCGGCGGATCTGGTGTTCTTCGACGGAACACTGTGGAGAGA
TGATGAAATGATTAGAGCGGGCCTGTCACCGAAATCAGGCCAAAGAATGGGCCACG
TCAGCGTGAACGACGCCGGAGGTCCAGTGGAGTGTTTCACGACGTGTGAAAAACC
ACGGAAGGTACTAATTCACATCAACAACTCCAACCCTATACTATTCGAGGACAGCCC
AGAGCGCAAGGACGTTGAAAGAGCGGGCTGGACAGTTGCGGAAGATGGCATGACA
TTTAGACTGGATACACC<u>ATG</u>ACGCTGCTGACACCGGACCAGCTCGAGGCTCAGTTG
CGACAGATAGGAGCAGAGAGGTACCACAACAGGCACCCTTTCCACCGCAAATTGC
ATGACGGCAAGCTTGACAAGGCCCAGGTGCAGGCTTGGGCACTCAACCGCTACTAC
TACCAGGCGCGCATTCCGGCGAAAGATGCGACACTGCTGGCGAGACTGCCGACAG
CGGAACTGAGAAGAGAATGGAGAAGAAGAATTGAAGATCATGATGGCACAGAACC
GGGCACAGGCGGCGTTGCGAGATGGCTAATGCTAACCGACGGACTTGGTCTAGACC
GTGACTACGTAGAGAGTCTAGATGGCTTGTTACCTGCCACTCGGTTCTCTGTCGACG
CTTACGTGAACTTTGTCCGTGACCAGTCGATACTAGCCGCCATCGCCAGTTCCCTTAC
TGAGCTCTTCAGCCCTACTATTATTTCGGAGCGCGTCAGCGGCATGCTACGACACTA
CGACTTTGTTTCGGAAAAAACATTGGCGTATTTCACTCCCAGACTGACACAAGCGCC

GAGAGATTCAGATTTCGCACTTGCGTATGTTAGAGAGAAGGCACGGACGCCAGAAC
AACAAAAAGAAGTGCTTGGAGCACTCGAGTTCAAGTGTAGCGTGTTATGGACGATG
CTCGACGCTCTGGATTATGCGTATGTTGAAGGCCATATTCCGCCGGGCGCGTTTGTTC
CATGACGGAAGCGCCGCATGTTGTTGCGGAAGGAACTGTGCTATCCTTCGCCCGAG
GACACCGGCTACAGCACGACCGAGTAAGGGACGTGTGGATAGTCCAAGCGCCGGA
GAAGGCATTCGTTGTTGAAGGCGCGGCACCACATATTCTACGTCTACTAGATGGGAA
GCGAAGTGTCGGAGAGATCATCCAGCAGTTGGCTATAGAGTTTAGCGCGCCGAGAG
AAGTTATTGCGAAAGATGTTCTGGCGCTGCTGTCAGAACTGACAGAGAAGAACGTG
CTGCATACATGACACTGCCGTCACCGCCGATGTCACTGCTGGCGGAACTGACACAT
AGATGCCCGCTGTCATGCCCGTATTGCTCAAATCCGCTGGAACTGGAAAGAAAGGC
GGCAGAGTTGGACACTGCGACATGGACAGCGGTTCTGGAACAGGCAGCCGAGCTA
GGCGTGTTACAGGTTCATTTCAGCGGAGGTGAGCCGATGGCGCGCCCTGACCTTGTA
GAGTTGGTCTCTGTAGCTCGCCGTCTTAACCTTTACTCTAACCTCATCACCTCAGGCG
TTCTGCTGGATGAACCGAAACTGGAAGCGCTGGATAGAGCGGGCCTGGATCATATT
CAACTGTCATTCCAGGACGTCACCGAGGCTGGCGCGGAAAGAATTGGCGGCCTGA
AAGGCGCGCAGGCACGTAAAGTCGCCGCCGCAAGACTCATCCGGGCCTCGGGCAT
CCCCATGACGTTAAACTTTGTCGTACACAGGGAAAATGTAGCTCGCATTCCGGAAAT
GTTTGCGCTGGCGAGAGAACTGGGCGCGGGCAGAGTTGAAATTGCGCATACACAAT
ATTATGGCTGGGGACTTAAGAATCGAGAAGCGCTGCTGCCGTCAAGAGATCAACTG
GAAGAATCAACAAGAGCGGTTGAAGCGGAGAGGGCTAAGGGAGGGCTATCAGTTG
ATTATGTTACACCGGATTATCATGCGGATAGACCGAAACCGTGCATGGGCGGCTGGG
GTCAGCGATTTGTTAATGTTACACCGTCAGGCAGAGTTCTGCCGTGCCACGCAGCAG
AAATTATTCCGGACGTCGCGTTTCCCAATGTCCAAGATGTAACATTATCTGAAATTTG
GAACATCTCGCCGTTGTTCAACATGTTCAGAGGCACGGACTGGATGCCCGAGCCTT
GTAGGTCTTGTGAGCGGAAGGAGAGAGATTGGGGCGGTTGTAGATGCCAAGCGATG
GCGCTGACAGGCAATGCGGCAAACACGGACCCTGTGTGTTCCCTTAGCCCATACCA
CGACCGAGTCGAGCAGGCAGTAGAGAACAACATGCAGCCGGAATCAACACTGTTC
TACCGCAGATATACATAAGAATTCGAGCTCCGTCGACAAGCTTGCGGCCGCACTCG
AGCACCACCACCACCACCACTGAGATCCGGCTGCTAACAAAGCCCGAAAGGAAGC
TGAGTTGGCTGCTGCCACCGCTGAGCAATAACTAGCATAACCCCTTGGGGCCTCTAA
ACGGGTCTTGAGGGGTTTTTTG
